# Seed micromorphology and calcium oxalate crystal characterization as taxonomic traits in selected species of the genus *Impatiens* L.

**DOI:** 10.1038/s41598-026-36206-w

**Published:** 2026-01-21

**Authors:** Agnieszka Rewicz, Justyna Polit, René Monzalvo, Monika Myśliwy, Maciej Studzian, Łukasz Pułaski, Saroj Ruchisansakun, Katarzyna Sanek, Kamil Najberek

**Affiliations:** 1 Department of Geobotany and Plant Ecology, University of Lodz, Banacha 12/16, Łódź, 90-237 Poland; 2 Department of Cytophysiology, University of Lodz, Pomorska 141/143, Łódź, 90-236 Poland; 3 School of Biological Sciences, Southern Illinois University, 1125 Lincoln Drive, Carbondale, IL 62901-6501 USA; 4 Institute of Marine and Environmental Sciences, University of Szczecin, Adama Mickiewicza 16, Szczecin, 70-383 Poland; 5 Department of Oncobiology and Epigenetics, Faculty of Biology and Environmental Protection, University of Lodz, Pomorska 141/143, Łódź, 90-236 Poland; 6 Laboratory of Transcriptional Regulation, Institute of Medical Biology PAS, Lodowa 106, Lodz, 93-232 Poland; 7 Department of Plant Science, Faculty of Science, Mahidol University, Ratchathewi, Bangkok, 10400 Thailand; 8 Institute of Nature Conservation, Polish Academy of Sciences, Al. Adama Mickiewicza 33, Kraków, 31-120 Poland

**Keywords:** Balsaminaceae, Biological invasions, Invasive alien species (IAS), Seed surface, Taxonomy, Ecology, Ecology, Evolution, Plant sciences

## Abstract

The study analyzed seeds of 12 *Impatiens* species from Europe, America, and Asia. Scanning electron microscopy (SEM), light microscopy, and confocal microscopy were used to analyze the micromorphology of the seed coat and the morphology of calcium oxalate crystals. A literature review revealed a lack of detailed carpological data for the studied species and a lack of information on the morphology of calcium oxalate crystals in seeds. The results revealed significant interspecific variation in seed surface characteristics, including seed shape, testal cell arrangement, and anticlinal and periclinal wall patterns. A wide variety of surface sculpture types was observed among the analyzed species. Furthermore, calcium oxalate crystals in the form of raphides were observed in all the studied species. The presence of raphides in species from three continents suggests that this may be a characteristic feature of the *Impatiens* genus, although it is insufficient to distinguish individual species.

**Supplementary Information:**

The online version contains supplementary material available at 10.1038/s41598-026-36206-w.

## Introduction

For years, plant species have primarily been described based on macroscopic morphological characters^1–3^. An integrative taxonomy approach can help resolve some uncertain or complex taxonomic cases^4,5^. Advances in molecular techniques and in mircroscopic imaging using scanning electron mircroscopy (SEM) have undoubtedly introduced new methods for determining the boundaries between organism groups^6,7^.

Floral traits are important in taxonomy and are widely used at all levels of the taxonomic hierarchy^8,9^. However, other traits, such as seeds and fruits, have also shown taxonomic significance in certain botanical families^10^. Historically, seed descriptions have mostly relied on simple visual observation, stereomicroscopy, or light microscopy, which in most cases failed to capture important structural features, leading to the undervaluation of seed variation^1–3^. Additionally, the availability of seed material has limited carpological research, as taxonomists generally focus on collecting plant specimens with flowers, or collections do not coincide with the fruit season. Presently, seed and fruit micromorphology studies are recognized as important in taxonomy, as documented in the literature^11–13^. SEM microscopy allows to visualize in detail many interesting features at different levels of the taxonomic hierarchy^14–16^. Nevertheless, the literature still lacks comprehensive and consistent practices in morphology that combine detailed anatomical descriptions of external and internal structures, including cross-sections and tissue composition which are crucial elements for precise species identification.

In Balsaminaceae, numerous studies highlight the significant role of seed shape and the micromorphology of the seed coat in solving taxonomic problems^17–20^ and reconstructing the systematics and evolution of species within the genus^16,21^. However, despite the widespread recognition of seed micromorphology as useful tool for species identification^22,23^, comprehensive and systematic studies across the entire family are still lacking. To date, fewer than 300 species of this family, representing less than 30% of its members, have been subjected to detailed SEM-based seed micromorphology studies^24,25^.

Calcium oxalate crystals are a notable structure found in seeds, with morphological variability depending on the plant group. These crystals play an important role in plant biology and can serve as helpful aids in species designation^26–29^. Calcium oxalate crystals have been identified in over 200 plant families^30,31^, occurring in most organs and tissues, including roots^32^, stems^33^, leaves^26^, floral structures^34^, and seeds^35^. Their morphology is highly diverse, appearing as styloids, prisms, druses, raphides, and crystal sand^34,36–39^. Individual plant species produce crystals with taxon-specific morphology and distribution, suggesting that their formation is not a random occurrence but rather a genetically regulated process^30,36,40,41^. Some studies have reported the presence of calcium oxalate crystals, including raphides and other morphologies, in various plant tissues across *Impatiens* species. Among these studies, Capacio and Belonias^42^ and Pimple et al.^43^ reported raphides in the leaves and stems of *I. balsamina.* On the other hand, Yang et al.^44^ identified rectangular crystals in the pollen grains of *I. balsamina*, and Pavlova and Glogov^45^ further confirmed the presence of raphides in the pollen of seven *Impatiens* species. Likewise, raphides within the tapetal cells of *I. niamniamensis*^46^ and *I. parviflora*^47^ were reported. Moreover, Mihai et al.^48^ described the occurrence of raphides in stem structures of *I. glandulifera*, suggesting that crystal formation is not limited to foliar or floral organs. Chen et al.^18^ mentioned the presence of calcium oxalate crystals in the stems, and Utami and Shimizu^17^ broadly suggested that many species within Balsaminaceae might contain such crystals.

However, regarding seeds, the literature offers only limited insights. Their results suggest that these crystals may have significant taxonomic importance. This raises the question of whether crystals are a constant character in the seeds of the genus and whether they take one or more morphological forms that can be used as diagnostic or secondary characters in taxonomy. Likewise, although the functional purpose of these microstructures in balsams is barely known, as some authors point out^17^, it is possible that they may share a great range of functions as reported in other species^49,50^ including among some of its identified functions; the mechanical protection against external agents, waste products storage, and regulators of calcium transport to seeds.

The present study aims to perform a meticulous analysis of the seed coat from selected representatives of the *Impatiens*. The research employs Scanning Electron Microscopy (SEM) and light microscopy to image the seed micromorphology and calcium oxalate crystals packages to assess the potential utility of these traits in the context of taxonomic classification.

## Materials and methods

### Studied species

We analyzed 12 species from the *Impatiens* genus, originating from three continents: North America (México), Europe (Croatia, Hungary, and Poland), and Asia (Thailand). Species identification was performed by the authors (see Author Contributions). Seeds were collected during 2021–2024 (in North America in 2021, in Europe between 2021 and 2024, and in Asia in 2021). They were placed in paper bags and then stored in a well-ventilated area. For each species, information on available carpological data was gathered (Table S1). The species nomenclature validation was adopted from The World Flora Online^51^. It should also be noted that two of the studied plants, namely *I. glandulifera* and *I. capensis*, are considered invasive alien species (IAS) and, because of the threat they pose to native biodiversity, are legally regulated in Poland. These restrictions include the need to obtain permission to use them. However, in the experiment performed, such permissions were not necessary, as seeds of these species were destroyed during SEM analyses, which met with the need for population control^52,53^. It should also be stressed that all seeds were destroyed after the analyses was completed to prevent the risk of dispersal. Consequently, no seeds were deposited in a publicly accessible herbarium.

### Scanning electron microscope (SEM) analyses

The seeds were sputter-coated with gold. Micro-morphological data were studied using a Phenom Pro X Scanning Electron Microscope at the Department of Invertebrate Zoology and Hydrobiology, University of Lodz, Poland. The seeds were fixed on brass tables and were sputter-coated with a 4 nm layer of gold. The observations were conducted at a magnification range of ×100–×3000 and with an accelerating voltage of 10 kV. Three dimensional models of the seed surface ultrastructure were generated using 3D Roughness Reconstruction software on the Phenom Electron Microscope. The SEM images were trimmed and arranged into plates.

### Biometric analyses of seeds

From each taxon, between 7 and 10 seeds were measured, depending on the availability of material. Observations and photographic documentation were performed using a Nikon SMZ-800 DS-Fi stereoscopic microscope equipped with a Coolview 2274 camera (Nikon). The seeds were analyzed for four quantitative traits: length, width, circumference, and surface area. The seed traits were automatically measured in the horizontal view using options in the biometric programme Cool View. All metric data are summarized in Table 1.

**Table 1:** Comparison of morphological characters of seeds of analyzed species from *Impatiens* genus.

Taxon	*N* seeds per species	Seed size (mm)	Seed shape	Colour	Primary ornamentation	Secondary ornamentation	Periclinal cell wall	Anticlinal cell wall
*Impatiens balfourii*	10	1.67 × 1.03/4.23/1.25	ellipsoid	dark brown	protrusive	digitiform	convex	irregularly curved
*Impatiens capensis*	10	3.64 × 2.72/9.54/5.63	ellipsoid	dark brown	protrusive	carinate	convex	irregularly curved
*Impatiens glandulifera*	7	4.21 × 3.42/12.22/9.99	subspheroid	grey-brown	reticulate	areolate	convex	irregularly curved
*Impatiens jiewhoei*	8	1.89 × 1.32/5.21/1.75	ellipsoid	yellow	protrusive	striate	convex	irregularly curved
*Impatiens kanburiensis*	9	4.32 × 3.32/12.24/9.21	ovate	dark brown	appendicular	threaded	convex	straight
*Impatiens longiloba*	7	3.74 × 1.83/9.57/5.34	ellipsoid	brown/dark brown	protrusive	digitiform	convex	irregularly curved
*Impatiens mexicana*	7	3.34 × 2.22/8.51/4.99	ellipsoid	dark brown	protrusive	carinate	convex	irregularly curved
*Impatiens noli-tangere*	10	3.43 × 2.54/8.79/4.89	ellipsoid	dark brown	protrusive	serrulate	convex	straight
*Impatiens parviflora*	10	3.23 × 2.14/9.42/4.98	ellipsoid	dark brown	protrusive	carinate	convex	straight
*Impatiens radiata*	7	2.31 × 1.71/6.52/2.46	ellipsoid	dark brown	protrusive	digitiform	convex	irregularly curved
*Impatiens spectabilis*	7	1.53 × 0.99/3.43/1.03	ellipsoid	orange/orange-brown	appendicular	threaded	convex	irregularly curved
*Impatiens suksathanii*	7	1.94 × 1.34/5.32/1.77	ellipsoid	brown/dark brown	protrusive	digitiform	convex	irregularly curved

### Light microscopy analyses and confocal imaging

Longitudinal and cross-sections, as well as isolated seed coats, were prepared from both dry and water-soaked seeds. Prior to sectioning, the water-soaked seeds were embedded in PBS-buffered 4% agarose. Thirty-micrometre sections of the seeds were prepared using a vibratome (Leica) and transferred onto SuperFrost^®^ slides. Observations and tissue analyses were conducted using a Nikon Eclipse E600W microscope. All images were captured using a DS-Fi1 CCD camera (Nikon). The location of crystal bundles was determined from cross-sectional images of seeds, and their density was estimated from images of isolated seed coats, counting bundles in five fields of view (1 mm²) for each species.

For confocal imaging, transverse cross-section (100 μm thick) were prepared using a vibratome (Leica). They were mounted on slides in glycerol and imaged using an LSM780 confocal microscope (Zeiss, Germany) equipped with an EC Plan-Neofluar 10×/0.3 objective. Images were acquired as 2 × 2 tiles in two fluorescence channels: 405 nm excitation/410–507 nm emission range (pseudocoloured blue in composite images) and 595 nm excitation/600–733 nm emission range (pseudocoloured red in composite images), as well as in transmitted light using a transmitted light photomultiplier without a pinhole. ZEN 2.1 Blue software (Zeiss) was used for image processing: deconvolution was applied as regularized inverse filter algorithm, individual images were stitched using Tiles module with 5% tile overlap.

### Statistical analyses

The following features were calculated: arithmetic average (x), maximum and minimum values (max and min), and the coefficient of variation for seed traits (Table S2). The crystal packet density values in Table 2 were further analyzed statistically. Differences between species were verified by ANOVA with Bonferroni correction and Tukey’s post-hoc test, as the Shapiro–Wilk test indicated normal distribution for all samples and Levene’s test confirmed homogeneity of variance. In contrast, differences between sections were verified by Kruskal-Wallis test and Dunn’s post-hoc test, because the Shapiro–Wilk test indicated deviations from normality. The software package STATISTICA PL. ver. 13.1 (Stat-Soft Inc. 2011) was used for all the numerical analyses.

**Table 2:** Location and density of crystal packets in the seeds of *Impatiens* species.

Taxon	Location of crystal packets	Crystal packets density (number per mm^2^)	Min	Max	SD
*Impatiens balfourii*	Large, thick-walled cells forming a single layer on the inner side of the exotesta	34	26.00	39.00	5.50
*Impatiens capensis*	Large, moderately thick-walled cells embedded in a layer of compressed parenchymatous mesotesta cells containing chloroplasts	26	21.00	31.00	3.96
*Impatiens glandulifera*	Large, moderately thick-walled cells pressed into a layer of compressed mesotesta cells around hilum area	5	2.00	7.00	1.95
*Impatiens jiewhoei*	Large, thick-walled cells forming a single layer on the inner side of the exotesta, characterized by a thick layer of suberin on the epidermis	44	38.00	53.00	6.11
*Impatiens kanburiensis*	Large, oval, thick-walled cells, often separated from each other, were loosely distributed in an area of compressed mesotesta cells	56	49.00	64.00	7.09
*Impatiens longiloba*	Large, moderately thick-walled cells on the inner side of the exotesta pressed into a layer of compressed mesotesta cells	22	19.00	26.00	2.70
*Impatiens mexicana*	Large, moderately thick-walled cells embedded in a layer of compressed parenchymatous mesotesta cells containing chloroplasts	33	29.00	37.00	3.65
*Impatiens noli-tangere*	Large, moderately thick-walled cells embedded in a layer of compressed parenchymatous mesotesta cells containing chloroplasts	6	4.00	10.00	2.61
*Impatiens parviflora*	Large, thick-walled cells on the inner side of the exotesta pressed into a layer of compressed mesotesta cells	12	8.00	15.00	3.03
*Impatiens radiata*	Large, thick-walled cells forming a single layer on the inner side of the exotesta pressed into a layer of compressed mesotesta cells	18	13.00	22.00	4.10
*Impatiens spectabilis*	In the layer that does not show a thickened wall and chloroplasts (mesotesta). Large, relatively thin-walled cells pressed into a layer of compressed mesotesta cells, characterized by a thick layer of suberin on the epidermis	22	18.00	25.00	2.88
*Impatiens suksathanii*	In the sclerenchymatous layer under the epidermis (mesotesta). Large, thick-walled cells forming a single layer on the inner side of the exotesta	24	19.00	27.00	3.44

## Results

### Seed surface micromorphology

**Fig. 1:**
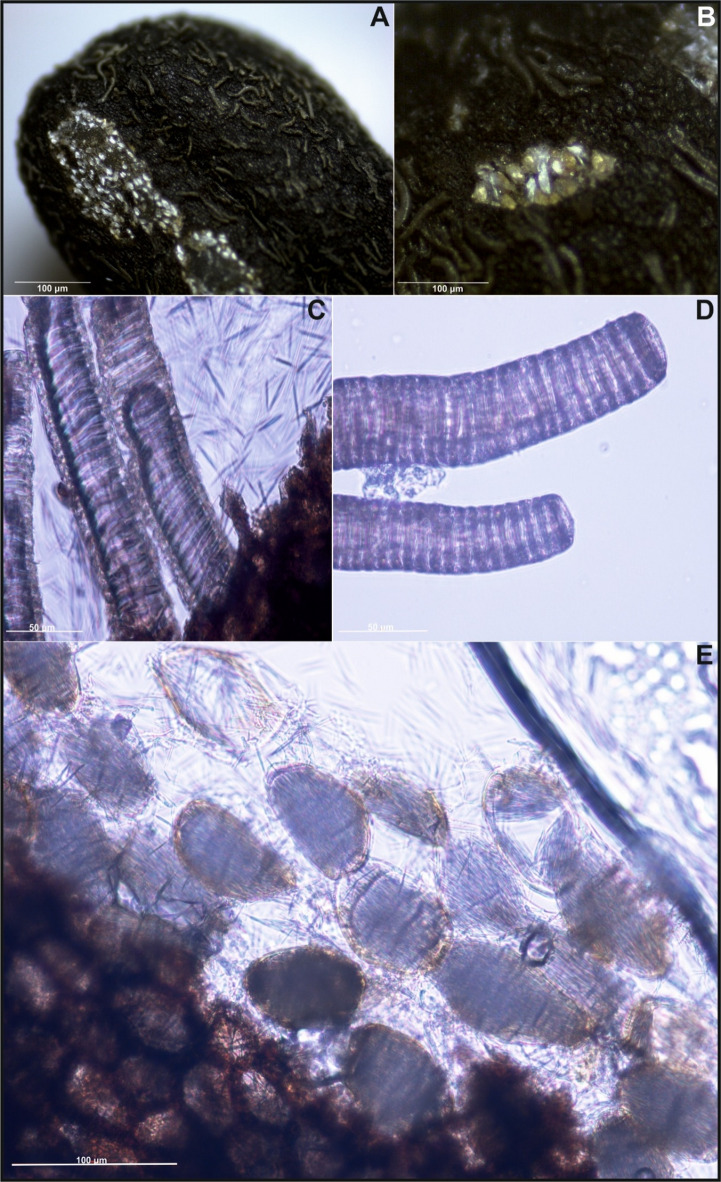
**(A***-***B**) View of the exposed crystal packets of *Impatiens kanburiensis* seed, (**C**-**D**) seed coat micromorphology under a light microscope, (**E**) crystal packages.

**Fig. 2:**
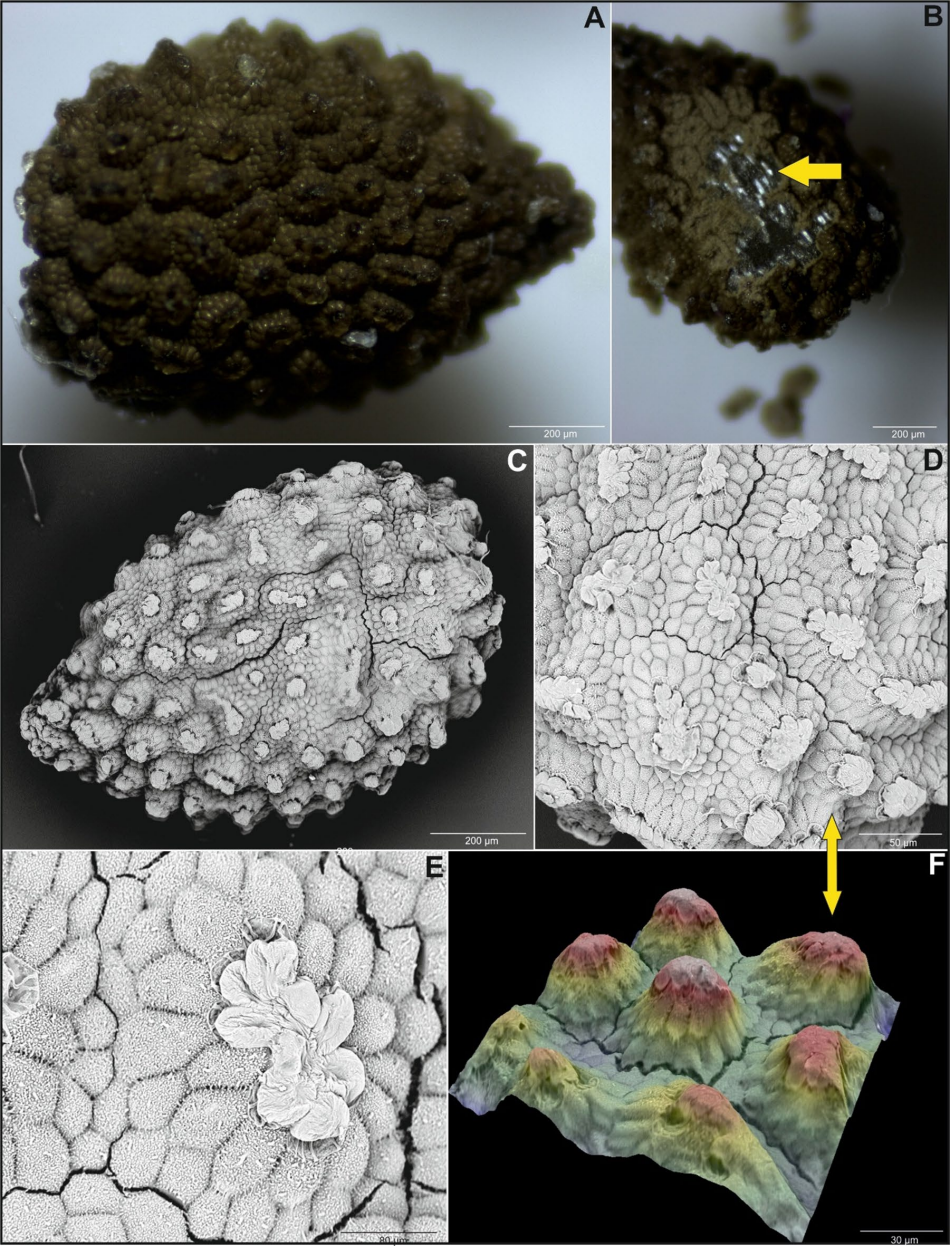
(**A**) General view of *Impatiens spectabilis* seeds under a light microscope, (**B**) a packet of crystals – marked with a yellow arrow, (**C**) view of seeds under SEM, (**D**-**E**) seeds micromorphology under SEM, (**F**) seeds surface 3D ultrastructure.

**Fig. 3:**
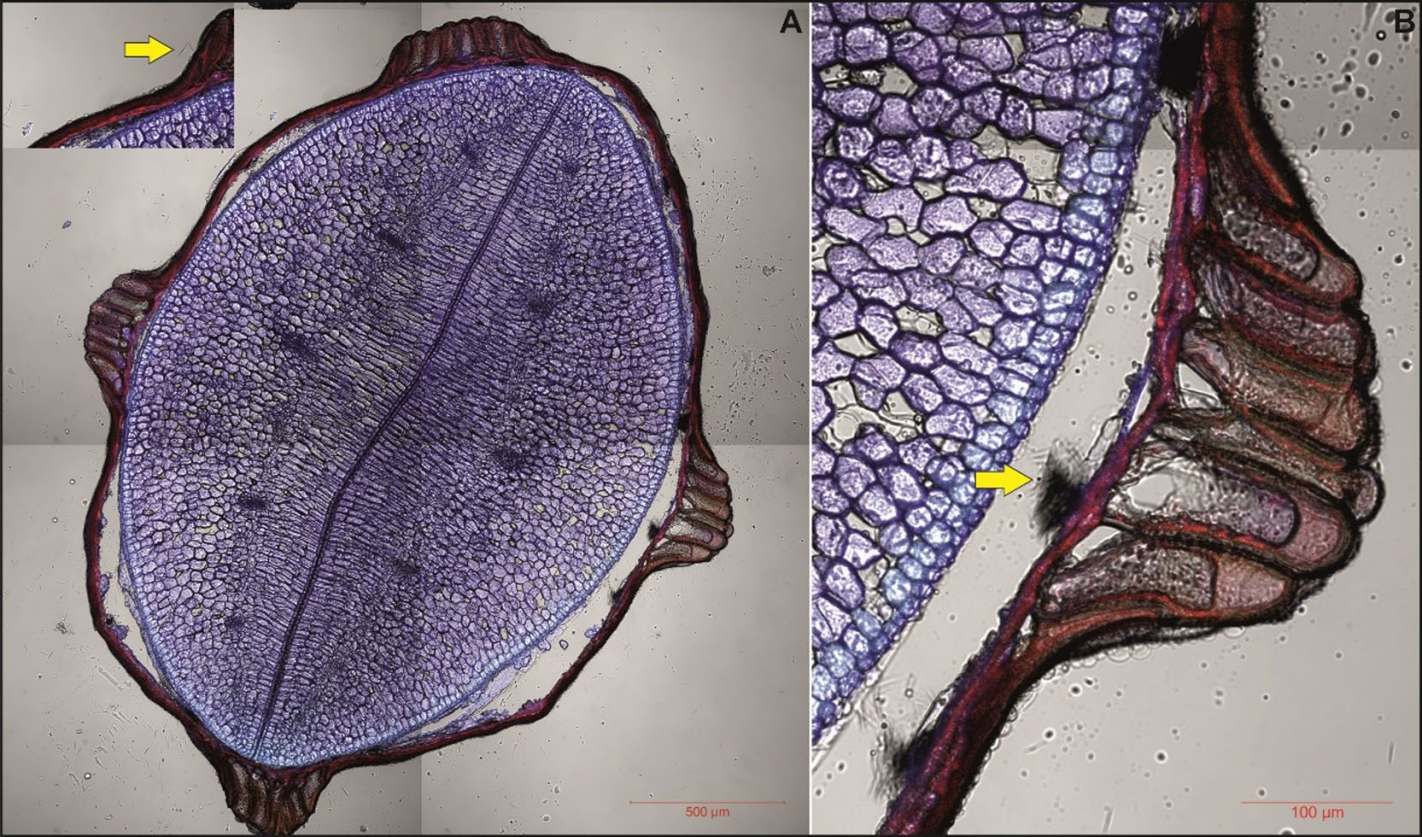
Representative confocal microscopy images of transverse cross-sections of *Impatiens capensis* seed. Autofluorescence in two fluorescence channels (for details see Materials and Methods section) is shown in blue and red color and is merged with transmitted light images. (**A**) whole seed cross-section, (**B**) enlarged fragment depicting exotesta – mesotesta boundary. Oxalate crystals are pointed by yellow arrows.

Most of the checked *Impatiens* species had elliptical seeds shape, except for *I. kanburiensis*, which had more rounded (ovate) seeds than the other species (see Figs. 1 and S1). Regarding seed ornamentation, the primary ornamentation in most species was of the protrusive type. However, *I. kanburiensis* (see Figs. 1 and S1) and *I. spectabilis* (see Fig. 2) had an appendicular type, and *I. glandulifera* had a reticulate type. Secondary ornamentation varied across species, with most showing a digitiform subtype, indicating that the protruding epidermal cells of the seed coat appeared finger-like. In the case of *I. kanburiensis* (see Fig. 1B–D, S1) and *I. spectabilis* (see Fig. 2A–C), the epidermal cell protrusions were filamentous or tubular, indicating a threaded subtype. On the other hand, four species—*I. noli-tangere*, *I. parviflora*, *I. mexicana*, and *I. capensis *shared similarities in both primary and secondary ornamentation (see Figs. 3, S3, and S4; Table 1). All these species also exhibited ridges on their seed surfaces. In *I. capensis*, there were four ridges evenly surrounding the seed, whereas the remaining species exhibited more than four ridges. In *I. noli-tangere*, the ridges were the most prominent, and they did not maintain continuity along the entire length of the seed (see Figure S4). Regarding periclinal cell walls, all analyzed species had convex cell walls, whereas anticlinal walls were mostly irregularly curved or straight (see Table 1). Two species (*I. spectabilis* and *I. jiewhoei*; see Figs. 2, S5, and S8) were particularly distinctive, characterized by a thick suberin layer on the epidermis. In the remaining species, the epidermis lacked suberin (see Table 2).

### Characterization of calcium oxalate crystals

**Fig. 4:**
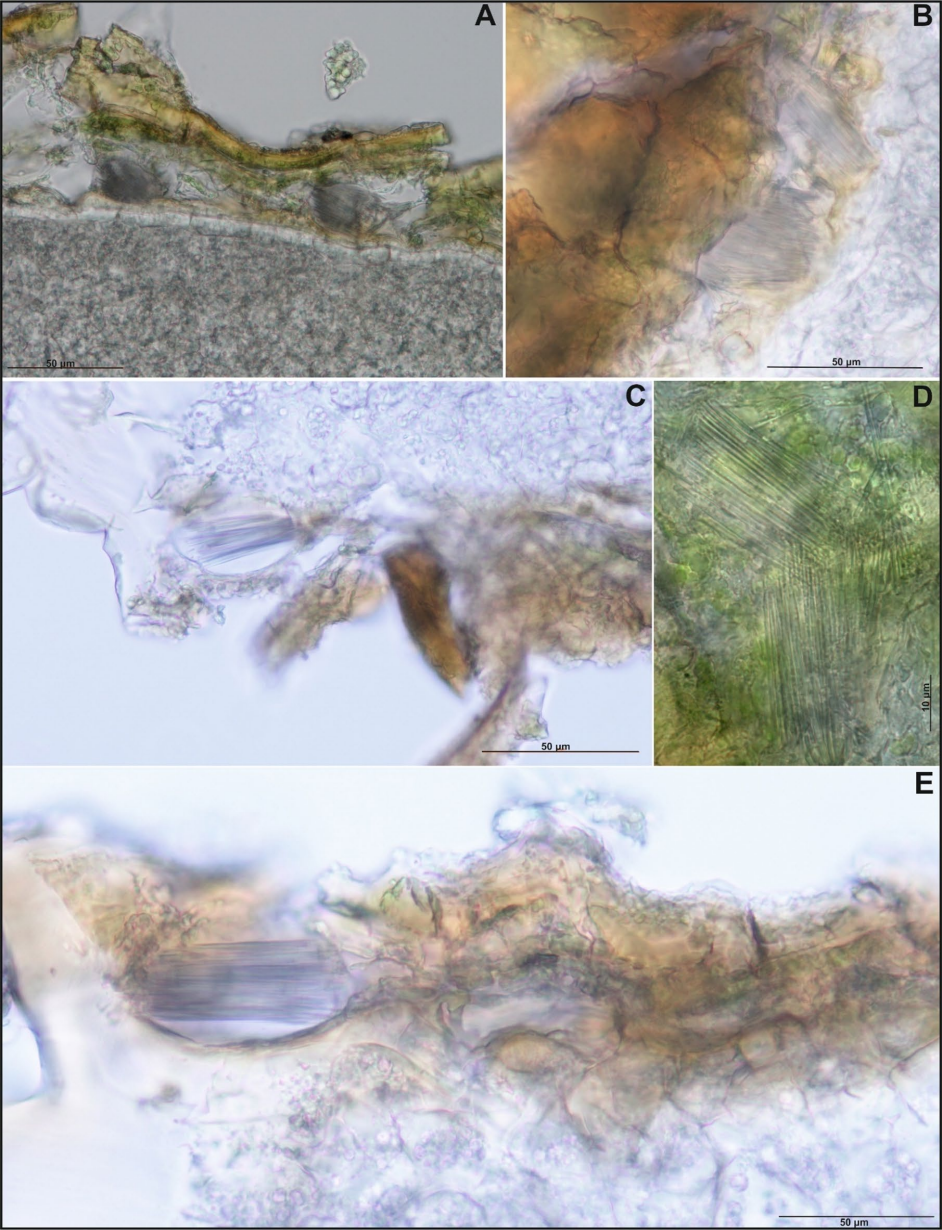
**(A**-**E**) Crystal packets in *Impatiens mexicana* seeds observed under a light microscope at various magnifications.

**Fig. 5:**
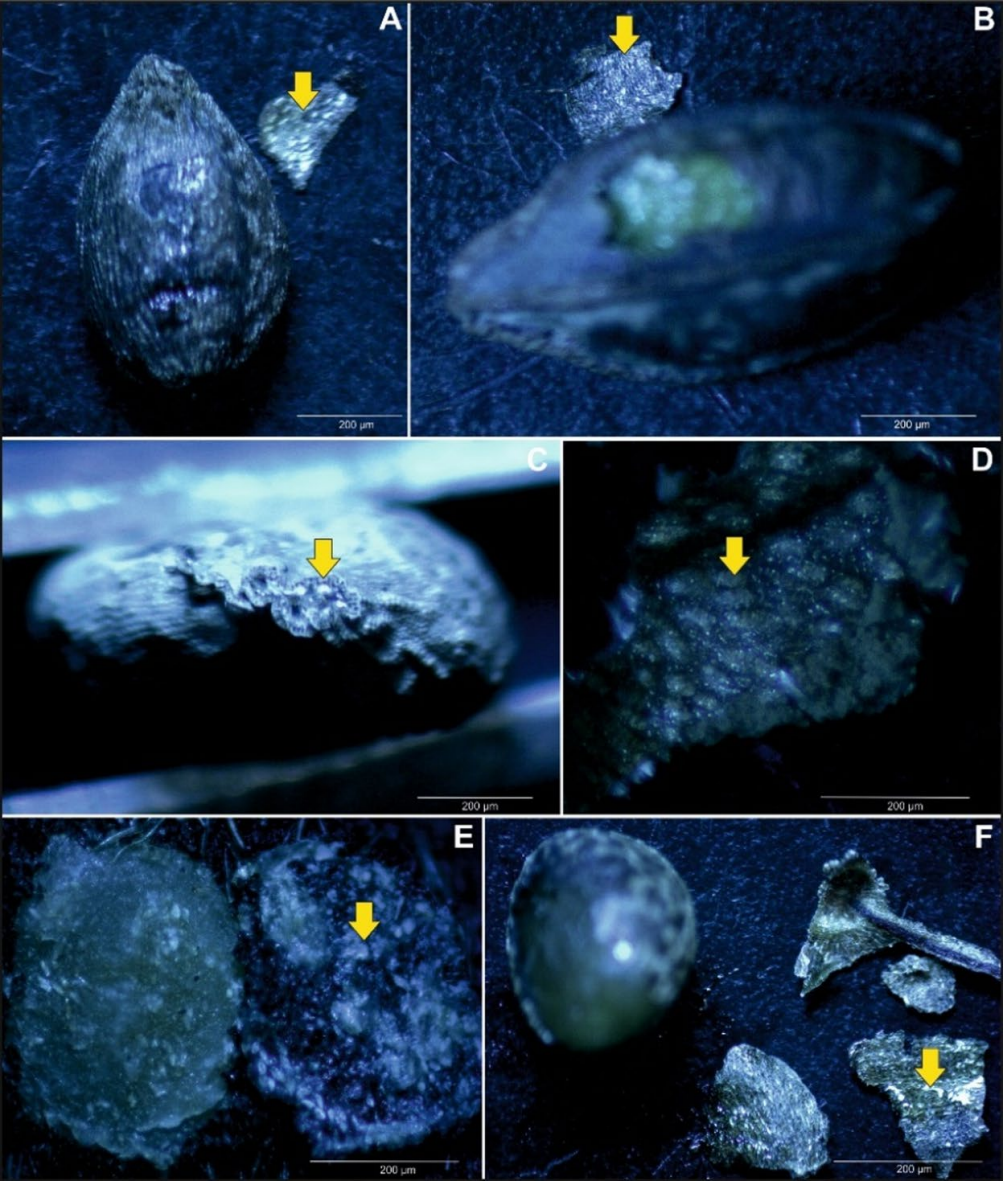
A packet of crystals observed under a light microscope; marked with a yellow arrow. (**A**) *Impatiens balfourii*, (**B**) *I. capensis*, (**C**) *I. glandulifera*, (**D**) *I. jiewhoei*, (**E-F**) *I. kanburiensis.*

Calcium oxalate crystals in the form of raphides, characterized by long, thin needle-like packet-forming structures, were observed in the seed coat (e.g., see Figs. 4, 5, S10, and S11). Crystal packets were located in large sclerenchymatous cells with lignified walls, positioned on the inner side of the exotesta and pressing into the compressed mesotesta cells (see Figs. 4, 5, and S12–S20). In *I. capensis*, *I. mexicana*, and *I. noli-tangere*, this mesotesta region contains parenchymatous cells with chloroplasts (e.g., see Figure S7). Only in *I. spectabilis* the crystal-containing cells do not exhibit strong wall lignification (see Figure S13). When the vacuolar structure was disrupted – such as by mechanical damage, the raphides were released, appearing as scattered needles under the microscope. This was visible especially in confocal images, where optical sectioning allowed for clear visualization the raphide bundles emerging from ruptured exotesta cells into the space which arose due to tearing of the exotesta from the mesotesta during slide preparation (see Fig. 3). In species such as *I. capensis*, *I. kanburiensis*, and *I. jiewhoei*, raphide bundles were visible after epidermal damage (see Figs. 1, 3, and S18). Although no significant differences in crystal morphology were observed among the studied species, variability was found in the frequency of cells containing raphide bundles and in their distribution within seed tissues across species (see Table 2).

Analysis of the number of crystal packets among the studied species revealed highly significant differences in their frequency of occurrence (ANOVA, *p* < 10^− 15^). Analysis of differences between species by Tukey’s post-hoc test allowed us to distinguish four groups, where the differences between any two species from different groups was significant at least at *p* < 0.05, while differences within each group were non-significant at this level (see Figure S22). These groups included: *I. kanburiensis* and *I. jiewhoei* with highest packet densities; *I. balfourii* and *I. mexicana* with high densities; the majority of species (*I. capensis*, *I. longiloba*, *I. radiata*, *I. suksathanii* and *I. spectabilis*) with moderate packet densities; and *I. parviflora*, *I. noli-tangere* and *I. glandulifera* with low packet densities. Interestingly, this classification did not align closely with systematic division of the genus into sections. Moreover, the only statistically significant differences between average crystal packet densities in sections (Dunn’s post-hoc test, *p* < 0.05) were between the section *Semeiocardium* and the remaining sections (*Impatiens* and *Racemosae*). In general, oxalate crystal packets were evenly distributed throughout the entire seed coat, but in *I. glandulifera* (the species with the lowest density), the crystals had a notably different spatial distribution, being located exclusively in the apical part of the seed (around the hilum).

## Discussion

### Taxonomic and ecological implications of seed coat morphology

Our results confirmed previous findings that the seed coat structure, with its diverse forms, is an important feature for interspecific identification within the genus *Impatiens*^16,54^. Our study provides comprehensive carpological descriptions of six previously unanalyzed *Impatiens* species under SEM: *I. jiewhoei*,* I. kanburiensis*,* I. longiloba*,* I. mexicana*,* I. spectabilis*, and *I. suksathanii.* We also incorporated information on six seed-described species from different populations: *I. balfourii*, *I. capensis*, *I. glandulifera*, *I. noli-tangere*, *I. parviflora*, and *I. radiata.* These species showed no morphological variability among populations, suggesting that the structure is stable in these taxa and corroborating previous studies^20,21,55,56^.

Three species—*I*. *capensis*, *I. mexicana*, and *I. noli-tangere*—all belong to the same section, *Impatiens.* They exhibit clear similarities in seed micromorphology, such as the presence of more or less distinct ribs. The greatest resemblance is observed between *I. capensis* and *I. noli-tangere*; in both, the ribs are straight but differ in number. This similarity results not only from their shared sectional affiliation and close phylogenetic relationships, but also from similar ecological requirements. Interestingly, phylogenetically distant species such as *I. parviflora* (sect. Racemosae) display seed ornamentation similar to that of *I. capensis* and *I. noli-tangere* (characterized by pronounced surface ribbing), which may support the hypothesis that seed traits are adaptive through convergent evolution. The remaining species in the Semeiocardium and Racemosae sections (Table S1) showed no similar patterns in seed microstructures. The morphological heterogeneity observed here indicates greater evolutionary divergence within these groups. This suggests distinct ecological adaptations and more dispersed phylogenetic relationships within the sections.

Analysis of seed sculptures from specific species indicated that some observed traits may have significant ecological and adaptive implications, including seed dispersal and interactions with the environment. An example of this is *I*. *capensis*, whose seeds are equipped with four distinct and strong ridges. According to previous studies, this species exhibits a high capacity to float in water, which may be due to ridges on its seeds^57,58^. This would be in line with the results of a previous experiment on the floating ability of *I. glandulifera* and *I. balfourii* seeds^55^, in which the authors suggested that a reduction in surface roughness likely decreased the seeds’ floating ability. Observing the seed structure of *I. capensis*,* I. mexicana*, and *I. noli-tangere*, which are genetically closely related species^59,60^, it can be noted that similar ridging occurs in both cases. In *I. noli-tangere*, the ridges are smaller, discontinuous, and occur in numbers exceeding four. While in *I. mexicana*, the ridges are interrupted, vary in height, and are often slightly wavy, without a clear linear arrangement. These differences may be related to the ecological functions of these species, particularly their dispersal strategies, but may also be considered a potential attribute for discriminating species.

It is also worth noting that *I. noli-tangere* can easily be mistaken for another ribbed species, *I. parviflora*. The fact that *I. noli-tangere* and *I. parviflora* are the only two *Impatiens* species naturally occurring north of 50° latitude – despite being evolutionarily distant and morphologically distinct in many respects – strongly suggests a case of convergent adaptation to comparable climatic or ecological pressures. Their shared latitudinal range may have driven the independent evolution of similar seed surface structures (e.g., pronounced ribs), perhaps to facilitate dispersal. Finally, another example of seed species with similar ecological functions but with sufficient morphological variation for separation was observed in *I*. *jiewhoei* and *I*. *spectabilis* (sect. Semeiocardium), characterized by a very thick epidermis that may serve a protective function, safeguarding the seeds from mechanical damage and pathogen attacks^31^.

The present study is a demonstration of effective application of various biological imaging techniques to carpology, including transmitted and reflected light microscopy and macroscopy, fluorescent microscopy (as confocal imaging), and scanning electron microscopy. Each of these methods provides unique insights into seed surface sculpture, arrangement of cells and tissues and biochemical composition of salient features (e.g., raphides or suberous layers), generating synergistic added value for full description of seed morphology and histology. The application of confocal imaging to elucidating seed structure has recently been recommended for plant developmental physiology^61^ – we demonstrate its potential for taxonomic aims.

### Roles of calcium oxalate crystals on interspecific taxonomical and structural variation of *Impatiens* spp.

The results of presented study revealed widespread presence of the calcium crystals in the form of raphides in the *Impatiens* species, while their location within the seed coat varied between species. This is also a potential attribute to use in discriminating species with similar morphological characters (e.g., in flowers), as in the case of *I. capensis* and *I. mexicana*. The exception of *I. glandulifera*, where raphides were observed only at the top of the seeds. These crystals are assigned various functions in plants. Calcium oxalate crystals may form within cells to maintain low levels of soluble and potentially toxic oxalic acid^30,36,40^. However, despite their widespread occurrence, their precise function remains incompletely defined and is still under discussion^62^. It is likely that calcium oxalate also serves as a regulatory reservoir for internal calcium stores^63,64^ or contributes to ion homeostasis, such as sodium and potassium balance^30^. Abundant presence of calcium oxalate raphides in some tropical species may indicate the increased need for calcium detoxification by sequestering in insoluble crystals, when growing in hot climate on calcium carbonate-rich soil. This is in line with a similar role being suggested for oxalate crystals in dry climate plants^65^. Moreover, calcium oxalate crystals may provide structural reinforcement for the seed coat and act as an effective defense mechanism against herbivory, deterring insects^31^ or grazing animals^66^ by irritating mucous membranes. This effect is particularly pronounced in crystals shaped as sharply pointed needles arranged in bundles^67,68^. Interestingly, we found out that raphides in *Impatiens* seeds were present in large idioblasts with strongly lignified cell walls, which is in line with the postulate that oxalate deposition and subsequent oxidation is a mechanism which serves to locally release hydrogen peroxide needed for molecular crosslinking of lignin^69^. Additionally, these crystals may play a role in regulating water absorption during germination initiation by inducing the rupture of seed coats as seeds swell and internal pressure increases, thereby facilitating radicle emergence^70^.

The observed diversity in the distribution and frequency of cells containing raphide bundles suggests that different species may utilize these structures in a species-specific manner, potentially related to their ecological adaptations. Among the species analyzed, particular attention is drawn to *I. glandulifera*, one of the most invasive plant species in Europe^71^. In our opinion, the presence of a small amount of crystals in the seeds of *I. glandulifera* may also have an impact on the high invasiveness of this species. In the case of plants containing a large amount of calcium crystals, such seeds are often less palatable to herbivorous animals, which limits their dispersal with feces. However, in the case of *I. glandulifera*, whose seeds contain only a small amount of crystals, concentrated mainly in the seed’s apex, the function of the crystals may be different (Figure S21). The low concentration of crystals makes the seeds more acceptable to ungulates, which promotes their consumption. Although seed consumption by animals is not widespread, seeds that pass through their digestive system may be released in new habitats. This mechanism facilitates seed dispersal over long distances, thereby promoting the colonization of new areas and increasing the invasiveness of *I. glandulifera*. In the case of livestock, it has been confirmed that *I. glandulifera* is consumed by sheep^72–74^. These animals are often grazed in several locations within a single locality, which may facilitate short-distance dispersal. However, grazing areas are sometimes relocated by over a dozen kilometers, for example when flocks are moved from higher mountain areas in autumn to pastures situated elsewhere. This may promote long-distance spread, since this balsam is flowering and setting seed during that period (Najberek, pers. commun.). In our opinion the presence of crystal packets in *Impatiens* seeds may affect their ability to float on water, facilitating the colonization of new habitats. In the case of *I*. *capensis*, the crystal packets form a sealing layer that enhances their buoyancy, allowing them to remain afloat for extended periods, even up to 200 days^75.^ This mechanism increases their chances of reaching new, favorable environments. Additionally, the characteristic structure of the seed coat, with four ribs, is reinforced by the crystals, which strengthen its structure^76^. These crystals may improve the stability and floating capabilities of the seeds, preventing them from sinking, thereby supporting the seed dispersal process in aquatic environments.

No differences in the shape of raphide crystals were found among the studied species. At the same time, the observed variations in the frequency of cells containing raphide bundles do not provide a basis for drawing conclusions about differentiation between species or determining the role of these structures in the taxonomy of the studied genus. These differences likely have a more subtle significance, related to interactions with local environmental conditions, rather than taxonomic differences that could be easily captured solely based on morphological or microscale characteristics. While the practical taxonomic significant of raphide count and distribution in *Impatiens* seeds is doubtful, it is important to note that the apparent convergent evolution of this trait in this genus (with e.g. sections *Racemosae* and *Impatiens* containing species with both low densities of raphides – *I. glandulifera* and *I. noli-tangere*, and high densities – *I. balfourii* and *I. mexicana*, respectively) shows significant similarities to the pattern of multiple evolutionary emergence of other seed features, such as threaded secondary ornamentation^16^. This shows that, given the known high genotype and phenotype plasticity in this rapidly evolving genus, calcium oxalate deposition is another important physiological adaptation available for *Impatiens*, and the biochemical and genetic underpinnings of differences in raphide patterns should be studied in greater depth.

## Conclusions

In the present study on the seed coat structures of twelve *Impatiens* species, valuable morphological information for identification is provided and confirms the reported trend that seed coat morphology is a reliable diagnostic feature within this genus. On the other hand, while the density of crystal packets in the studied species showed non-phylogenetic but ecological affinities, further studies across more species of the genus are imperative to verify this pattern. Our findings may offer new insights into the functions of certain seed structures in specific species, such as external ridges and calcium oxalate crystals. Their significance is likely substantial, as these structures may be associated with species’ dispersal abilities and their potential to colonize new areas. For a detailed study of crystals to assess their taxonomic importance, it is necessary to use methods that visualize their morphological configurations.

## Supplementary Information

Below is the link to the electronic supplementary material.

Supplementary Material 1

## Data Availability

All data supporting the findings of this study are included in the manuscript and its supplementary material.
