# Supplementary material for: Seed micromorphology and calcium oxalate crystal characterization as taxonomic traits in selected species of the genus Impatiens L
Source: Sci Rep. 2026 Jan 21;16:5884. doi: 10.1038/s41598-026-36206-w (PMC12894852; doi:10.1038/s41598-026-36206-w)
Supplement: Supplementary file 1 — Supplementary Material 1 [file 41598_2026_36206_MOESM1_ESM.pdf]

SUPPLEMENTARY MATERIAL

TABLES

Table S1. Studied material of particular *Impatiens* species with corresponding sampling localities. Available literature on seed morphology for each species is also included. The division of sections follows Yu et al. (2015).

	**Taxon**	**Section**	**Sampling localities (country, state, code)**	**Literature data**
1	*Impatiens balfourii* Hook. f.	Racemosae	Croatia; Zagreb, Istria, ID6, Hungary, Veszprém-Balaton, IDIB2	Song et al. 2005; Najberek et al. 2020
2	*Impatiens capensis* Meerb.	Impatiens	Poland; Podgrodzie, Lubin, Unin, Police	Rewicz et al. 2020
3	*Impatiens glandulifera* Royle	Racemosae	Poland; Cracow, Muszyna, Rajsko, Zakopane	Najberek et al. 2020; Abid et al. 2011; Maciejewska-Rutkowska and Janczak 2016
4	*Impatiens jiewhoei* Triboun & Suksathan	Semeiocardium	Thailand; Kanchanaburi, Mueang Kanchanaburi District, S. Ruchisansakun 1788	No data
5	*Impatiens kanburiensis* T.Shimizu	Semeiocardium	Thailand; Kanchanaburi, Sai Yok District, S. Ruchisansakun 1785	No data
6	*Impatiens longiloba* Craib	Racemosae	Thailand; Chiang Mai, Chom Thong District, S. Ruchisansakun 1806	No data
7	*Impatiens mexicana* Rydb.	Impatiens	México; Agua Blanca de Iturbide, Hidalgo IMMEX004-23, IMMEX005-23, IMMEX006-23	No data

8	*Impatiens noli-tangere* L.	Impatiens	Poland; Sieradz, Lodz	Song et al. 2005, 2022; Utami and Shimizu 2005; Chen et al. 2007; Jin et al. 2008
9	*Impatiens parviflora* DC.	Racemosae	Poland; Sieradz, Lodz, Zakopane, Olsztyn	Song et al. 2005
10	*Impatiens radiata* Hook.f.	Racemosae	Thailand; Chiang Mai, Chom Thong District, S. Ruchisansakun 1805	Zhang et al. 2016
11	*Impatiens spectabilis* Triboun & Suksathan	Semeiocardium	Thailand; Kanchanaburi, Thong Pha Phum District, S. Ruchisansakun 1771 SR 1779 (33)	No data
12	*Impatiens suksathanii* Ruchis. & Triboun	Semeiocardium	Thailand; Kanchanaburi, Thong Pha Phum District, S. Ruchisansakun 1772	No data

Table S2. Comparison of biometric traits of seeds in the analyzed species of *Impatiens*: X -arithmetic average , Min, Max - maximum and minimum values, SD - standard deviation.

**Species**	**Length**				**Width**				**Circuit**				**Area**			
	**X**	**Min**	**Mak**	**SD**	**X**	**Min**	**Mak**	**SD**	**X**	**Min**	**Mak**	**SD**	**X**	**Min**	**Mak**	**SD**
Impatiens balfourii	1.67	1.49	1.86	0.14	1.03	0.95	1.19	0.09	4.24	4.00	4.41	0.13	1.25	1.01	1.58	0.15
Impatiens capensis	3.64	3.20	3.94	0.28	2.72	2.36	3.00	0.20	9.54	8.71	9.92	0.43	5.63	5.12	6.28	0.44
Impatiens glandulifera	4.21	3.44	4.80	0.46	3.41	3.11	3.74	0.25	12.21	11.43	12.89	0.47	9.99	9,69	10.28	0.23
Impatiens jiewhoei	1.89	1.22	2.33	0.36	1.32	1.02	1.61	0.20	5.21	4.57	6.52	0.63	1.75	1.22	2.26	0.36
Impatiens kanburiensis	4.32	3.54	4.53	0.31	3.32	3.01	3.58	0.19	12.24	9.50	13.73	1.25	7.43	5.42	9.36	1.09
Impatiens longiloba	3.75	3.41	4.51	0.40	1.84	1.09	2.40	0.45	9.57	8.61	11.07	0.87	5.34	4.51	6.44	0.76
Impatiens mexicana	3.34	2.21	4.14	0.75	2.22	1.99	2.71	0.27	8.51	6.91	9.81	1.10	5.00	4.24	5.80	0.62
Impatiens noli-tangere	3.43	3.10	3.79	0.20	2.54	2.19	2,85	0,23	8.79	8.02	9.92	0.69	4.90	4.36	5.48	0.36
Impatiens parviflora	3.23	2.98	4.02	0.35	2.15	1.59	2.46	0.26	9.42	8.09	11.21	1.10	4.98	4.39	5.67	0.48
Impatiens radiata	2.31	2.00	3.19	0.41	1.71	1.40	2.27	0.32	6.52	5.19	7.34	0.79	2.46	2.04	3.30	0.50
Impatiens spectabilis	1.53	1.02	2.18	0.45	0.99	0.91	1.09	0.05	3.43	3.09	4.20	0.37	1.03	0.91	1.24	0.11
Impatiens suksathanii	1.94	1.38	2.58	0.43	1.34	1.24	1.44	0.07	5.33	4.60	6.10	0.56	1.77	1.14	2.50	0.49

FIGURES

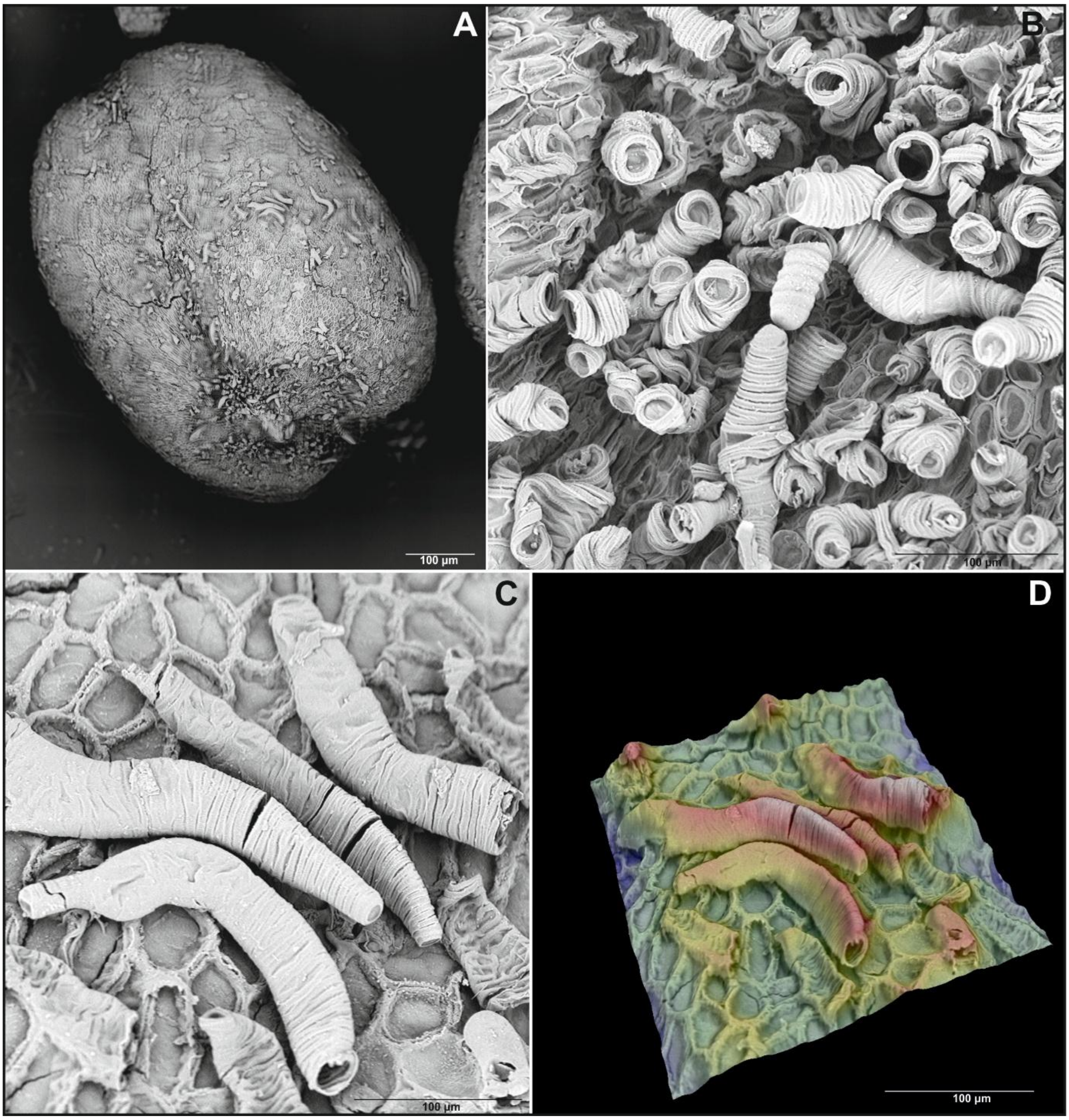

Figure S1. A) General view of *Impatiens kanburiensis* seeds under SEM, B-C) seed coat micromorphology, D) seeds surface 3D ultrastructure.

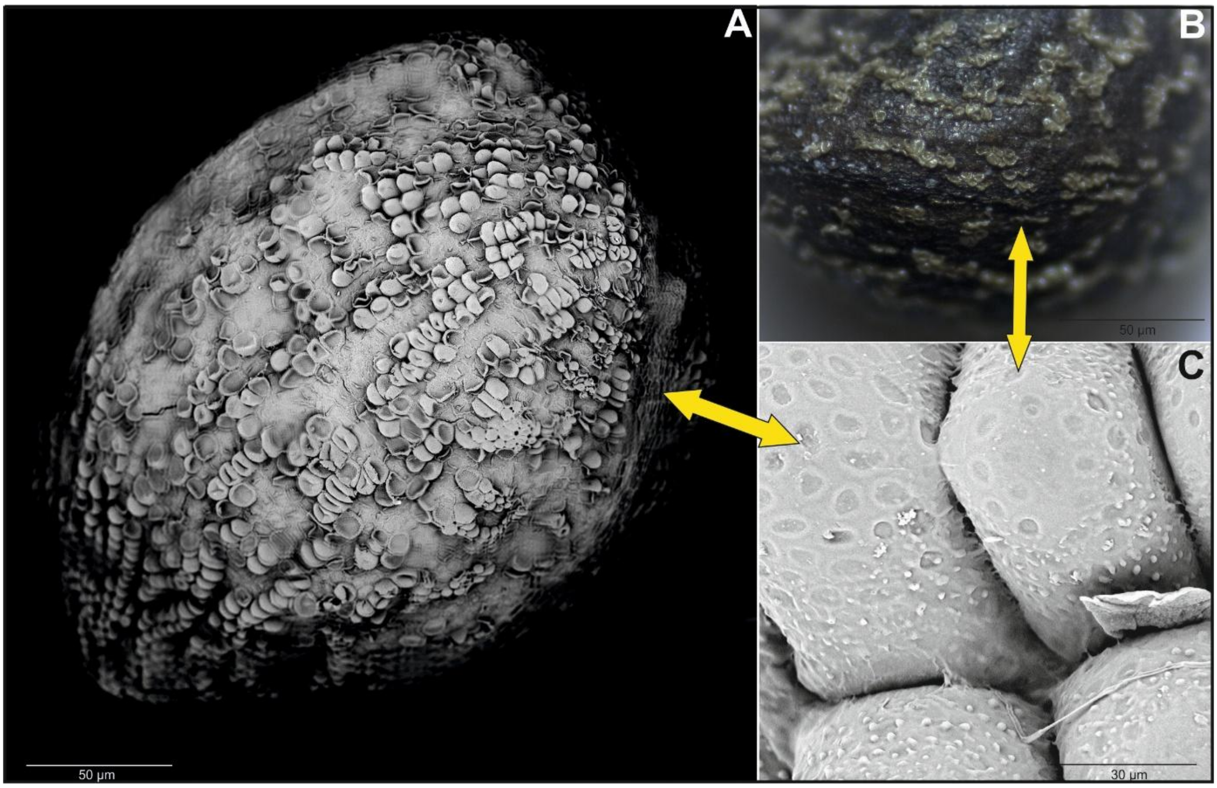

Figure S2. A) General view of seeds of *Impatiens longiloba* under SEM, B-C) seeds micromorphology, marked with a yellow arrows.

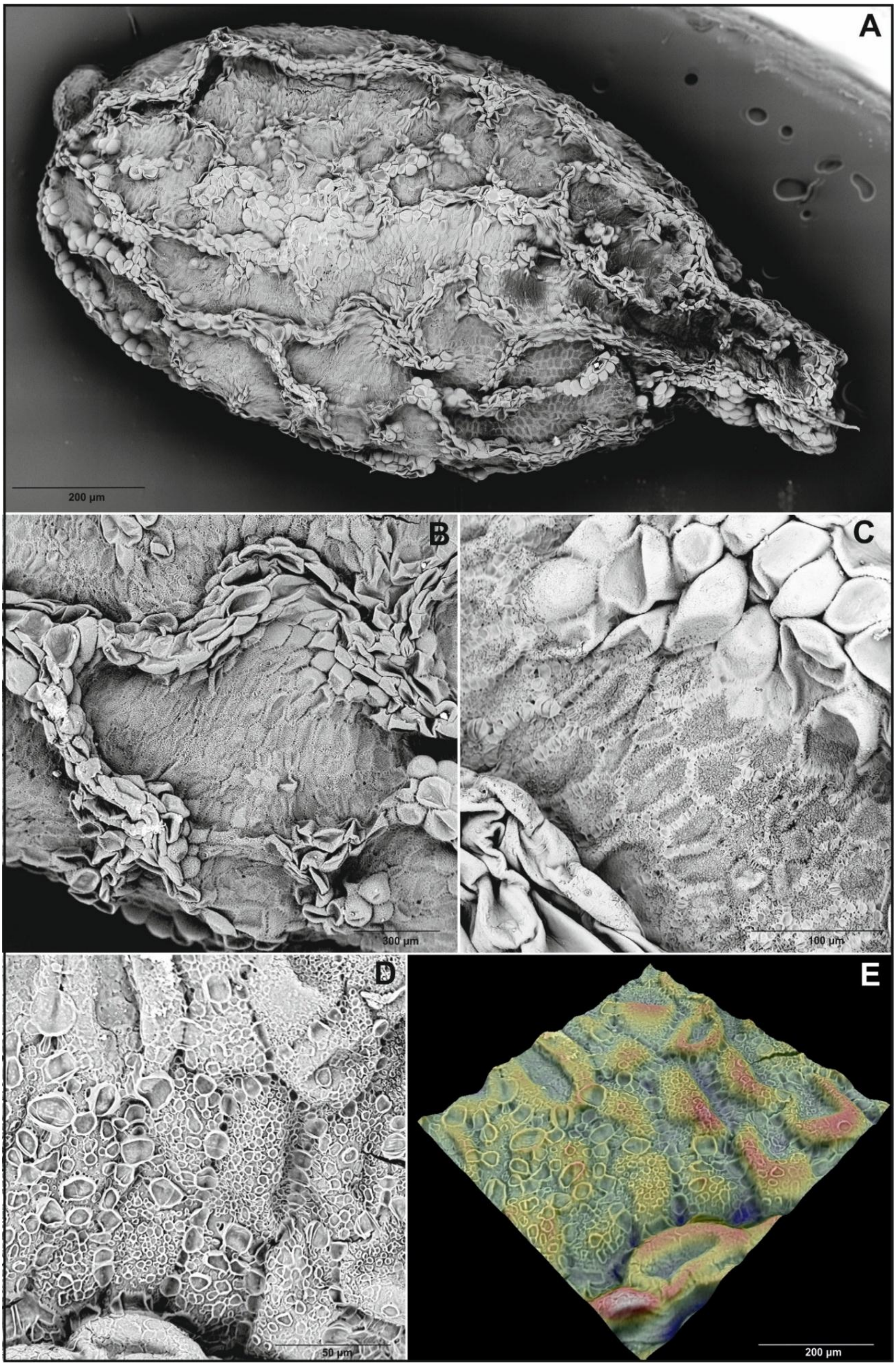

Figure S3. A) General view of *Impatiens mexicana* seeds under SEM, B-D) seeds coat micromorphology under SEM, E) seeds surface 3D ultrastructure.

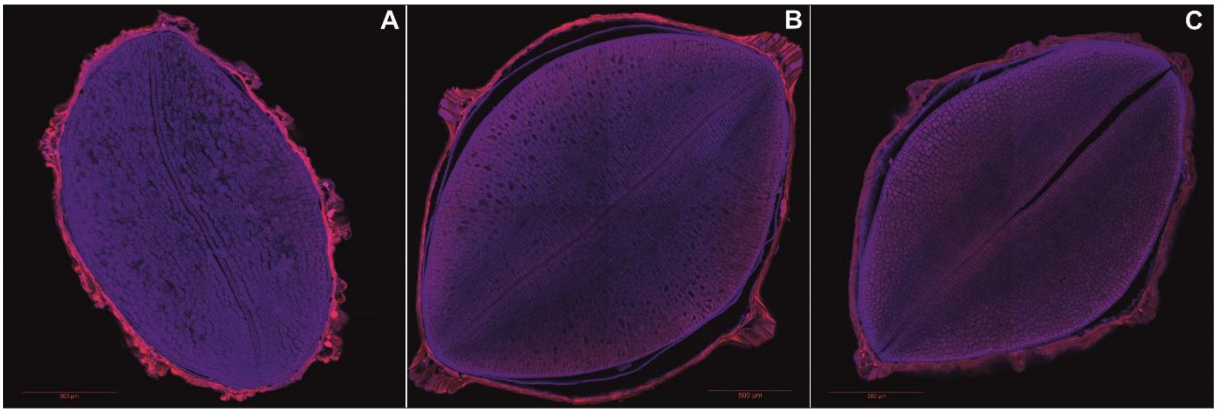

Figure S4. Representative confocal microscopy images of transverse cross-sections of A) *Impatiens mexicana*, B) *I. capensis*, C) *I. noli-tangere* seeds. Autofluorescence in two fluorescence channels (for details see Materials and Methods section) is shown in blue and red color.

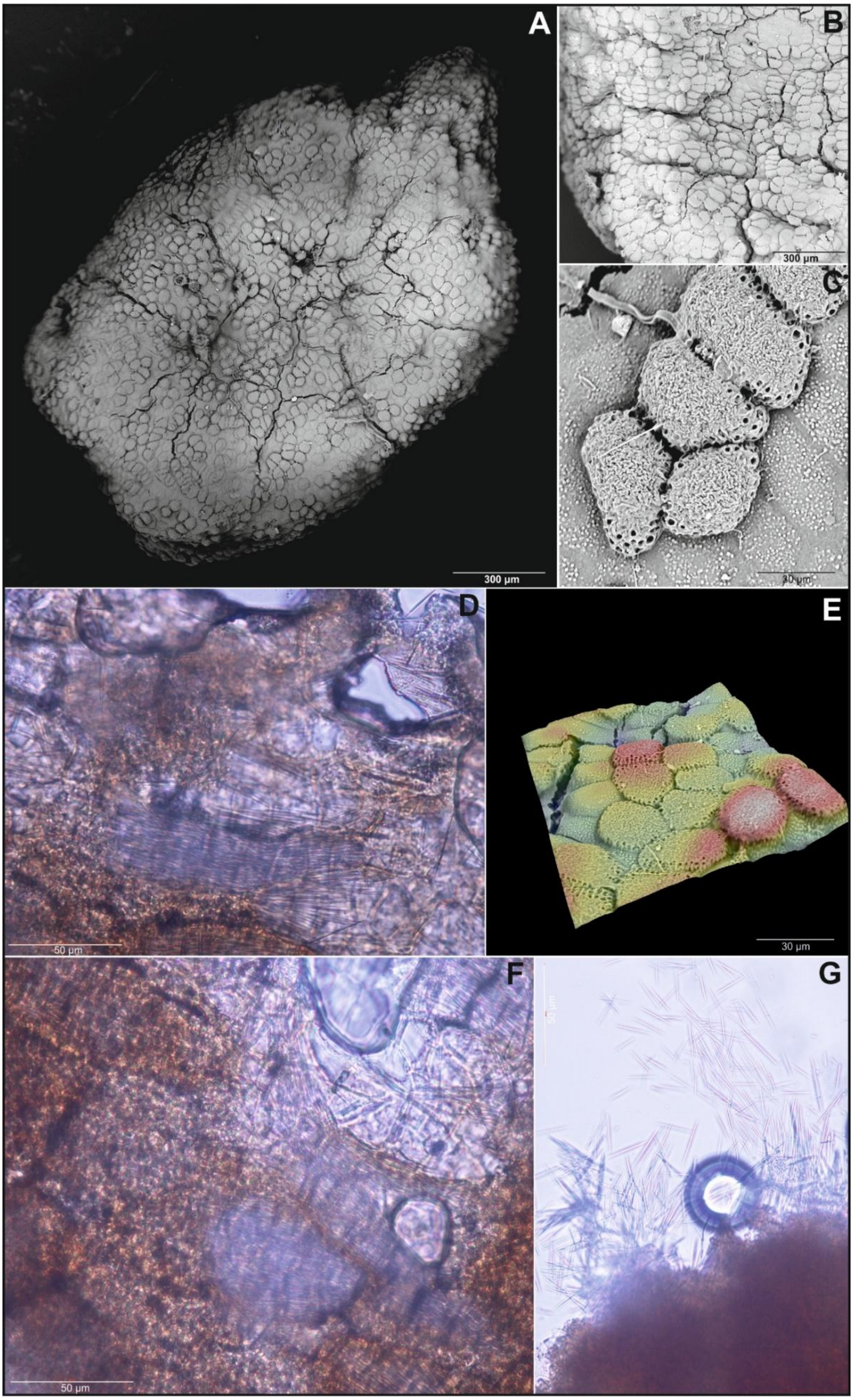

Figure S5. A) General view of seeds of *Impatiens jiewhoei* under SEM, B-C) seeds coat micromorphology under SEM, D, F) crystal packages under a light microscope, E) seeds surface 3D ultrastructure, G) torn crystal packet with visible scattered crystals.

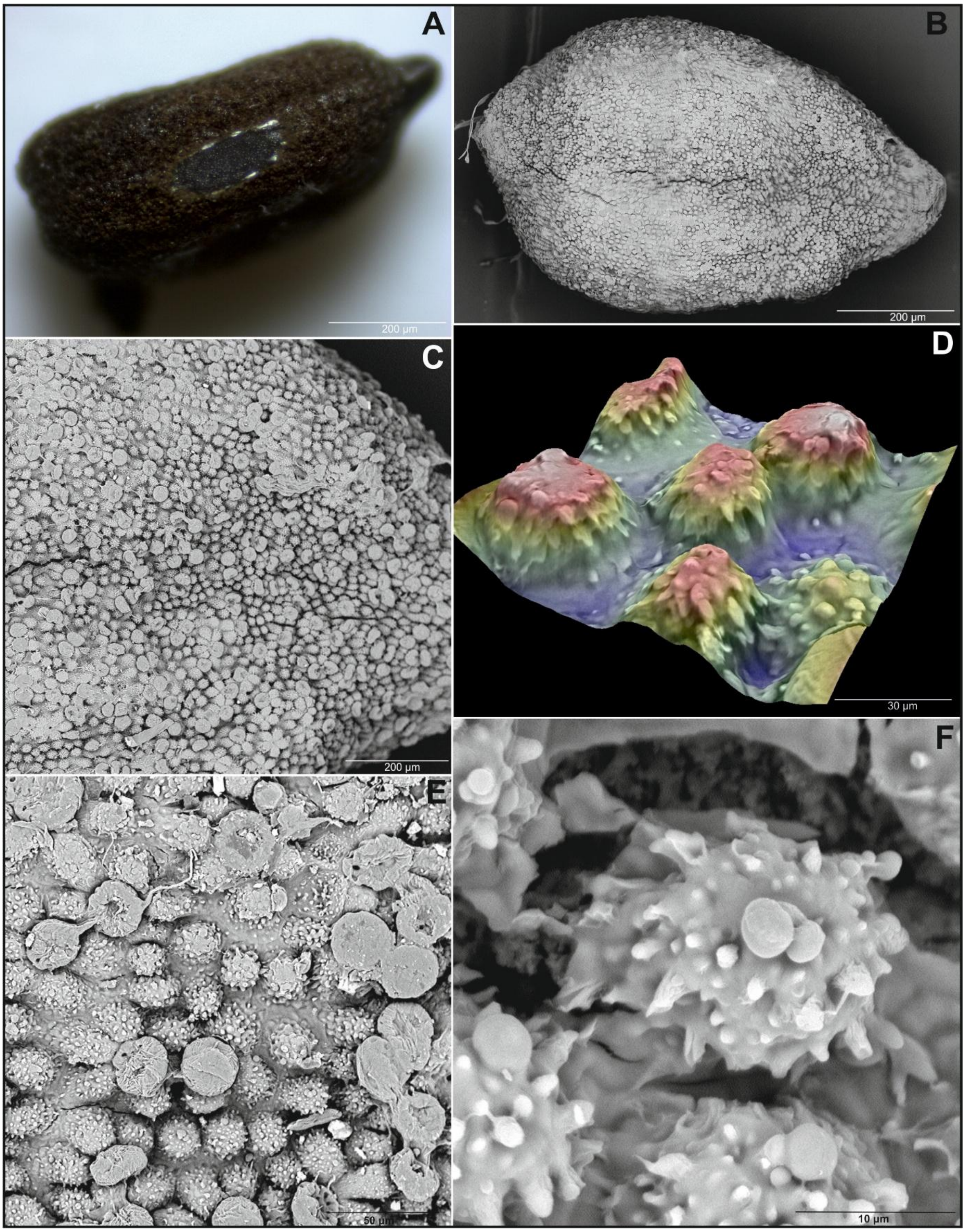

Figure S6. A) General view of *Impatiens suksathanii* seeds under a light microscope, B) general view of *I. suksathanii* seeds under SEM, D) seeds surface 3D ultrastructure, C, E, F) seeds coat micromorphology under SEM.

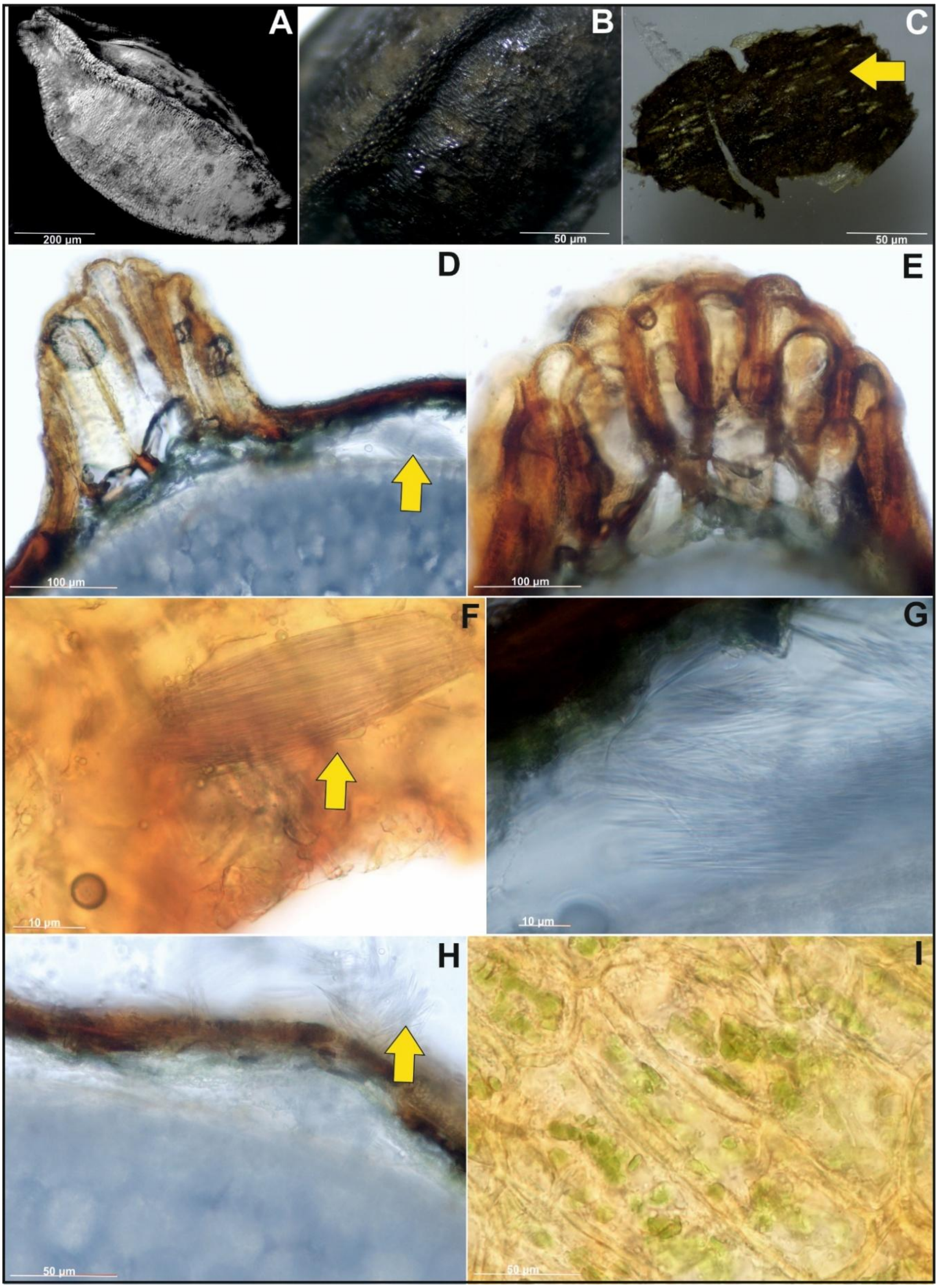

Figure S7. A) General view of seed of *Impatiens capensis* under SEM, B) view of the rib under a light microscope, C, F, G) crystal packages, marked with a yellow arrows, D, E) cross section through the rib, H) torn crystal packet with visible scattered crystals, I) parenchyma tissue with visible cells containing chlorophyll under a light microscope.

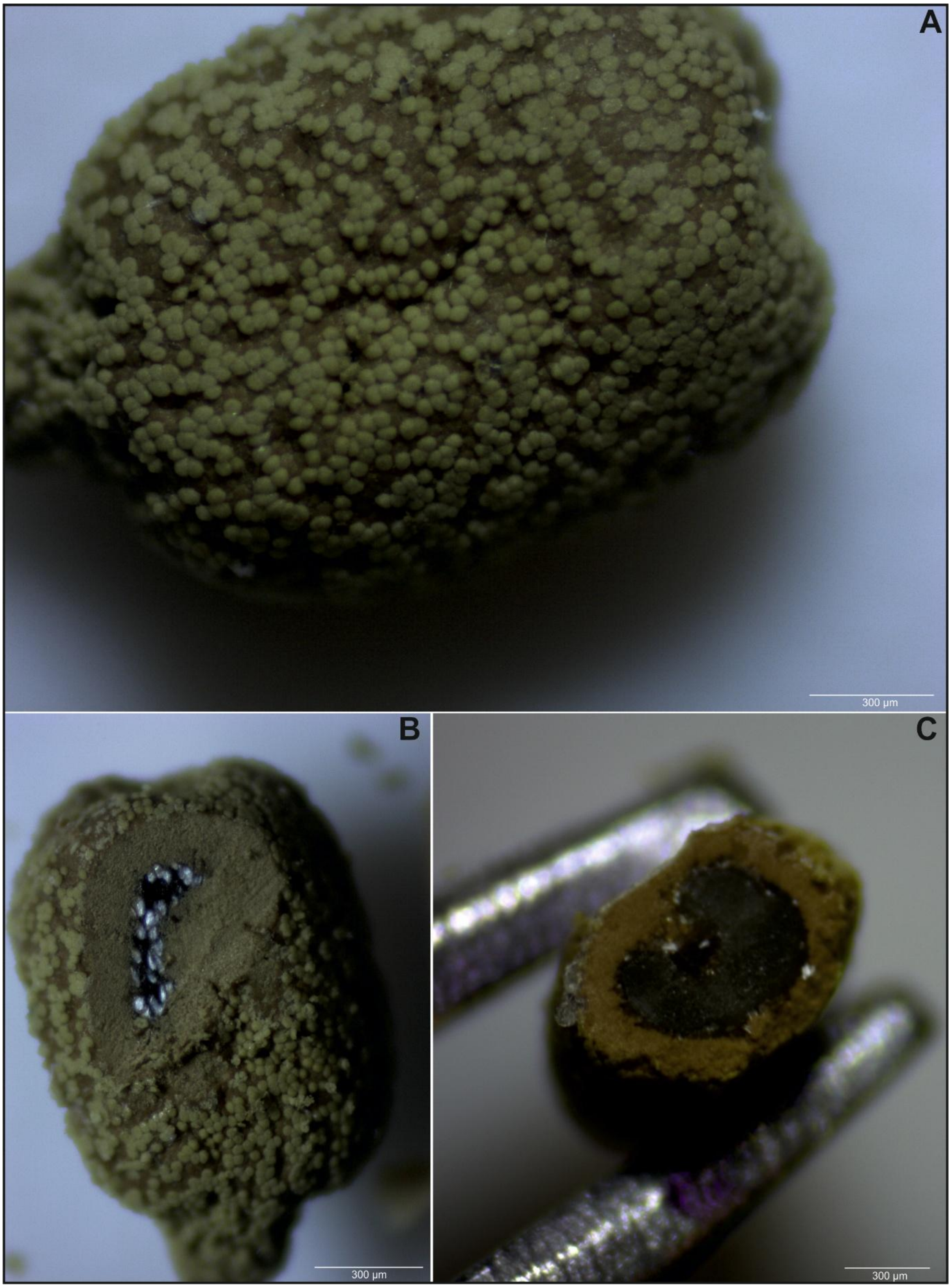

Figure S8. A) The view of the seeds from a light microscope of *Impatiens jiewhoei*, B) view of the exposed crystal packets, C) cross section of the seeds.

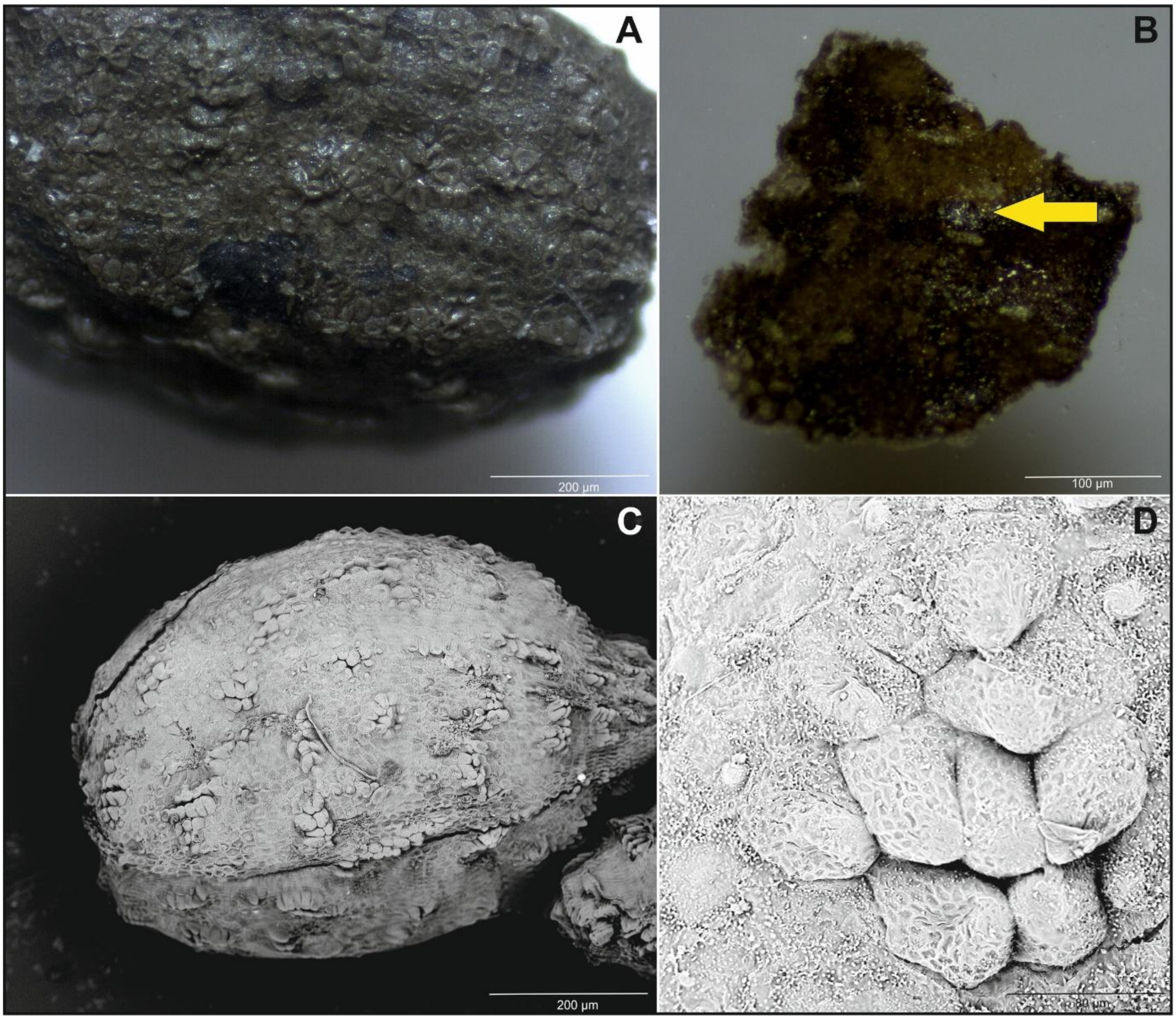

Figure S9. A, D) Seeds micromorphology under light microscope (A) and SEM (D), B) crystal packages under light microscope, marked with a yellow arrow, C) general view of *Impatiens radiata* seeds under SEM.

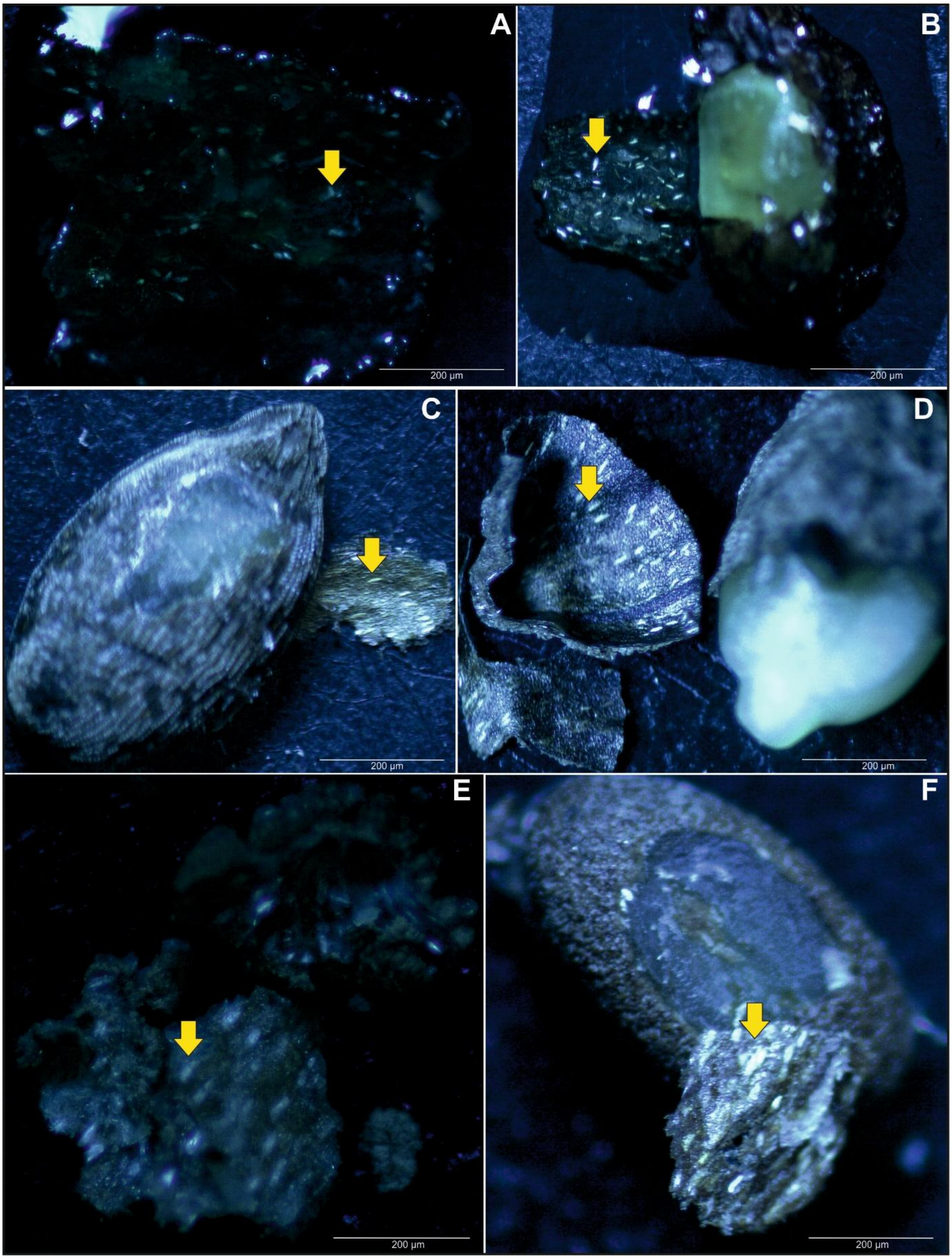

Figure S10. A packet of crystals observed under a light microscope; marked with a yellow arrows, A) *Impatiens mexicana,* B) *I. noli-tangere,* C) *I. parviflora,* D) *I. radiata,* E) *I. spectabilis,* F) *I. suksathanii*.

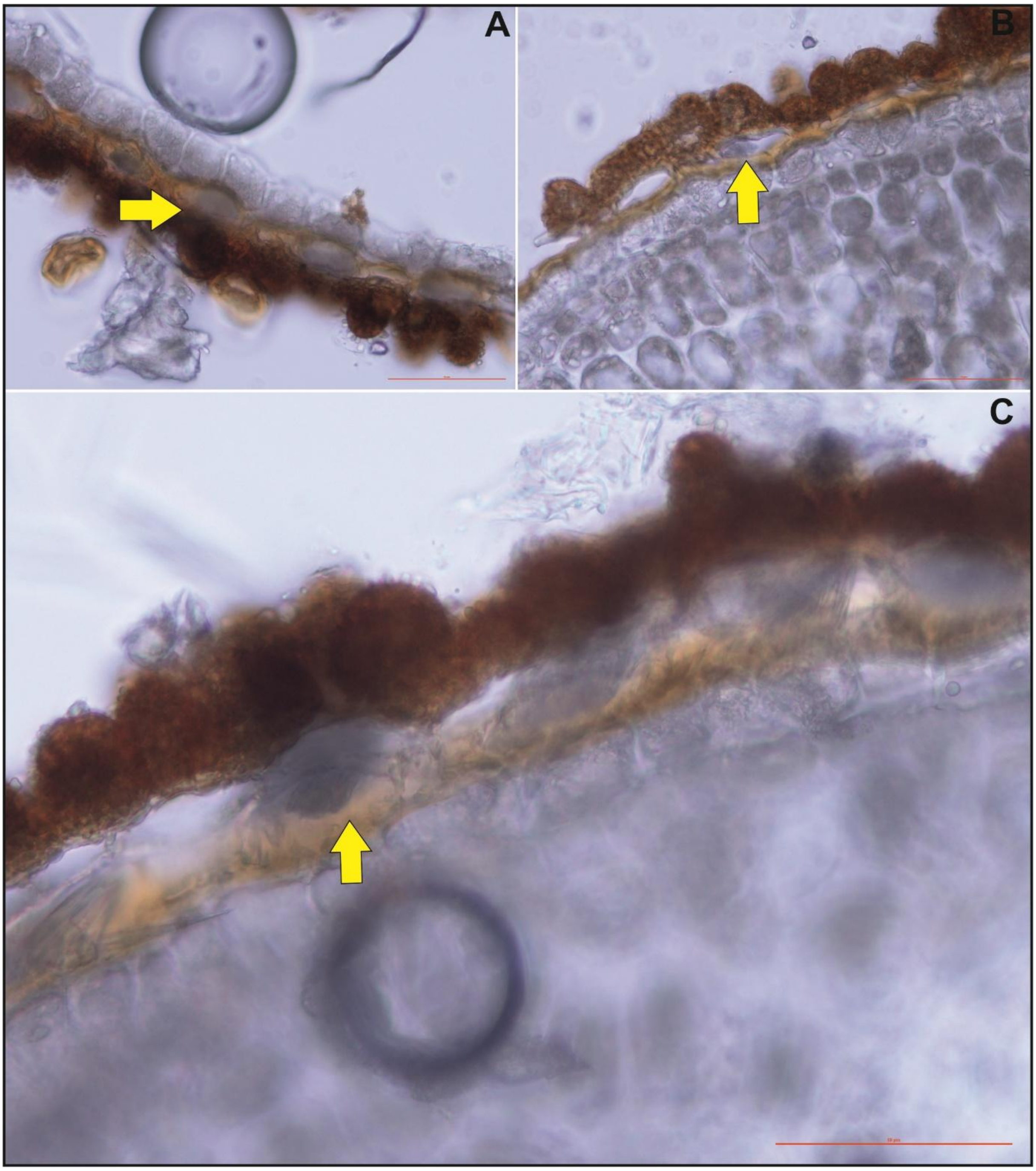

Figure S11. A-C) A packet of crystals observed under a light microscope in *Impatiens suksathanii* seeds observed at different magnifications; marked with a yellow arrows.

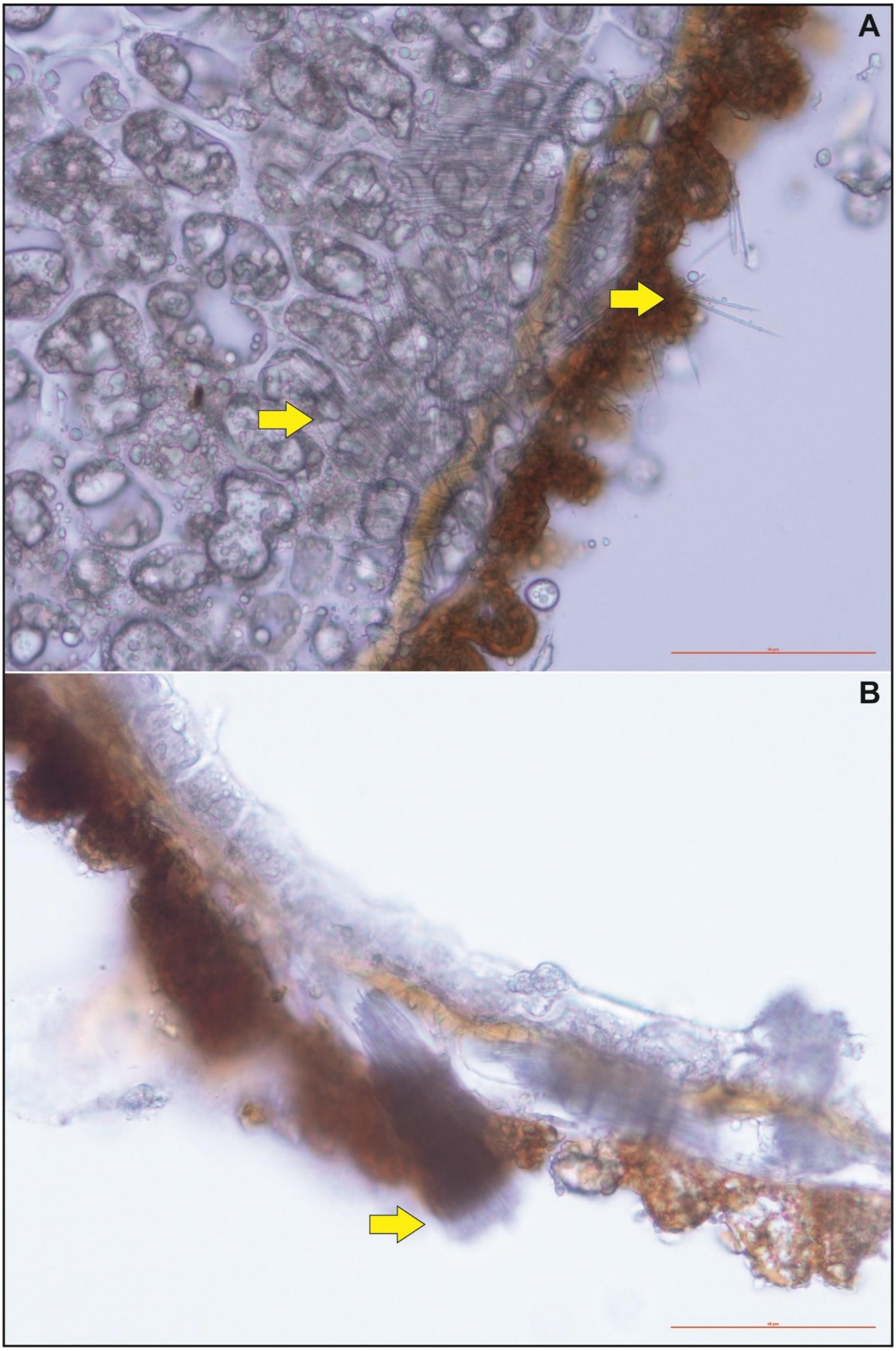

Figure S12. A-B) A packet of crystals observed under a light microscope in *Impatiens suksathanii* seeds observed at different magnifications; marked with a yellow arrows.

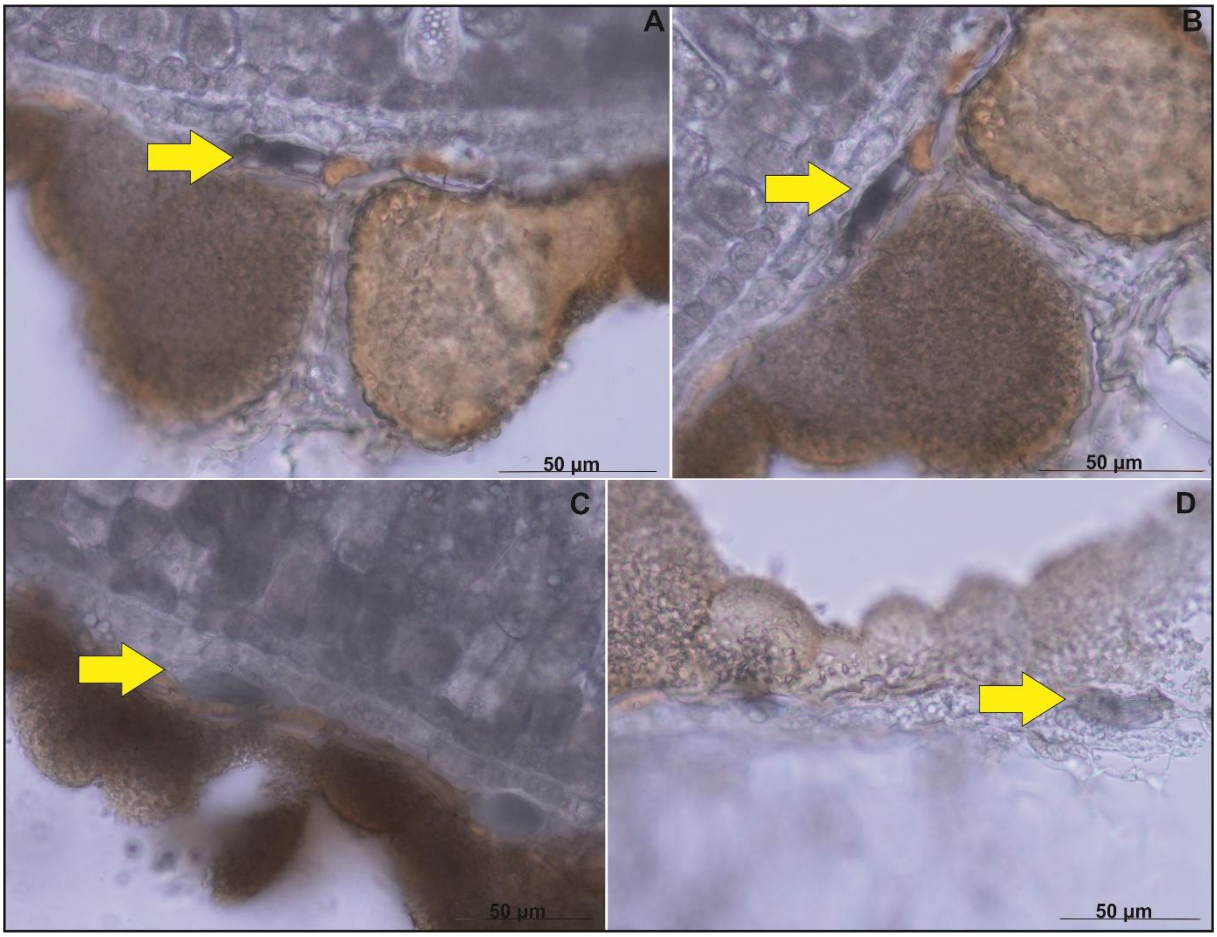

Figure S13. A-D) A packet of crystals observed under a light microscope in *Impatiens spectabilis* seed observed at different magnifications; marked with a yellow arrows.

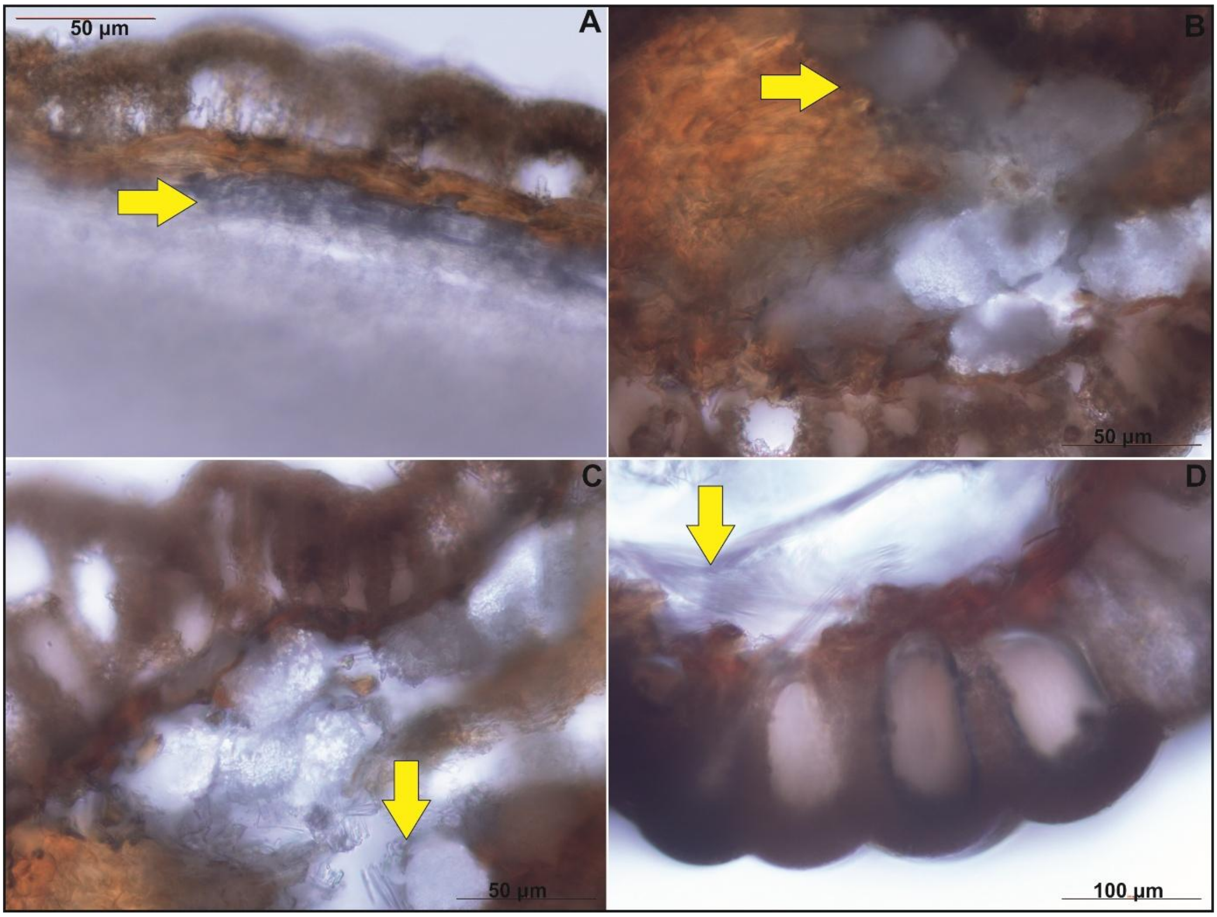

Figure S14. A-D) A packet of crystals observed under a light microscope in *Impatiens glandulifera* seeds observed at different magnifications; marked with a yellow arrows.

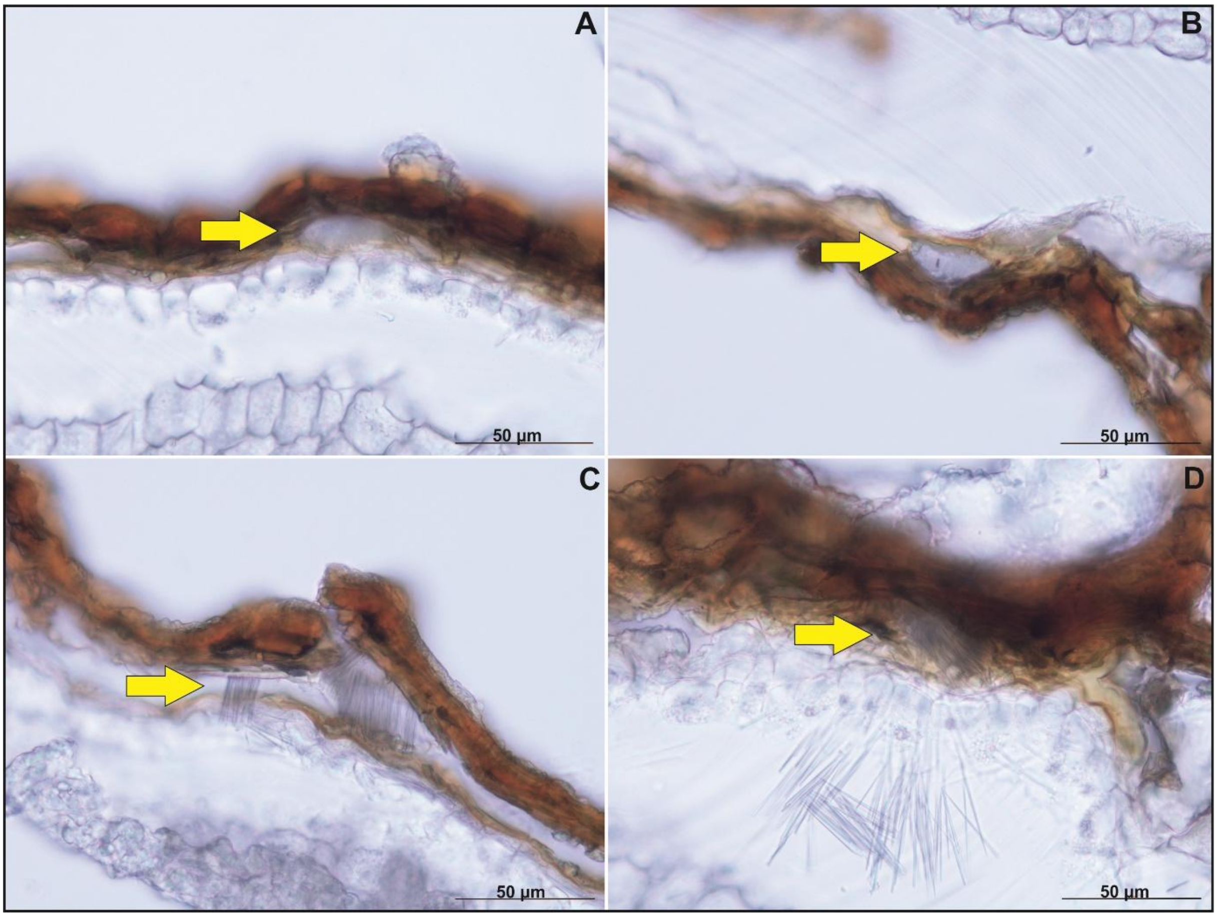

Figure S15. A-D) A packet of crystals observed under a light microscope in *Impatiens noli-tangere* seeds observed at different magnifications; marked with a white arrows.

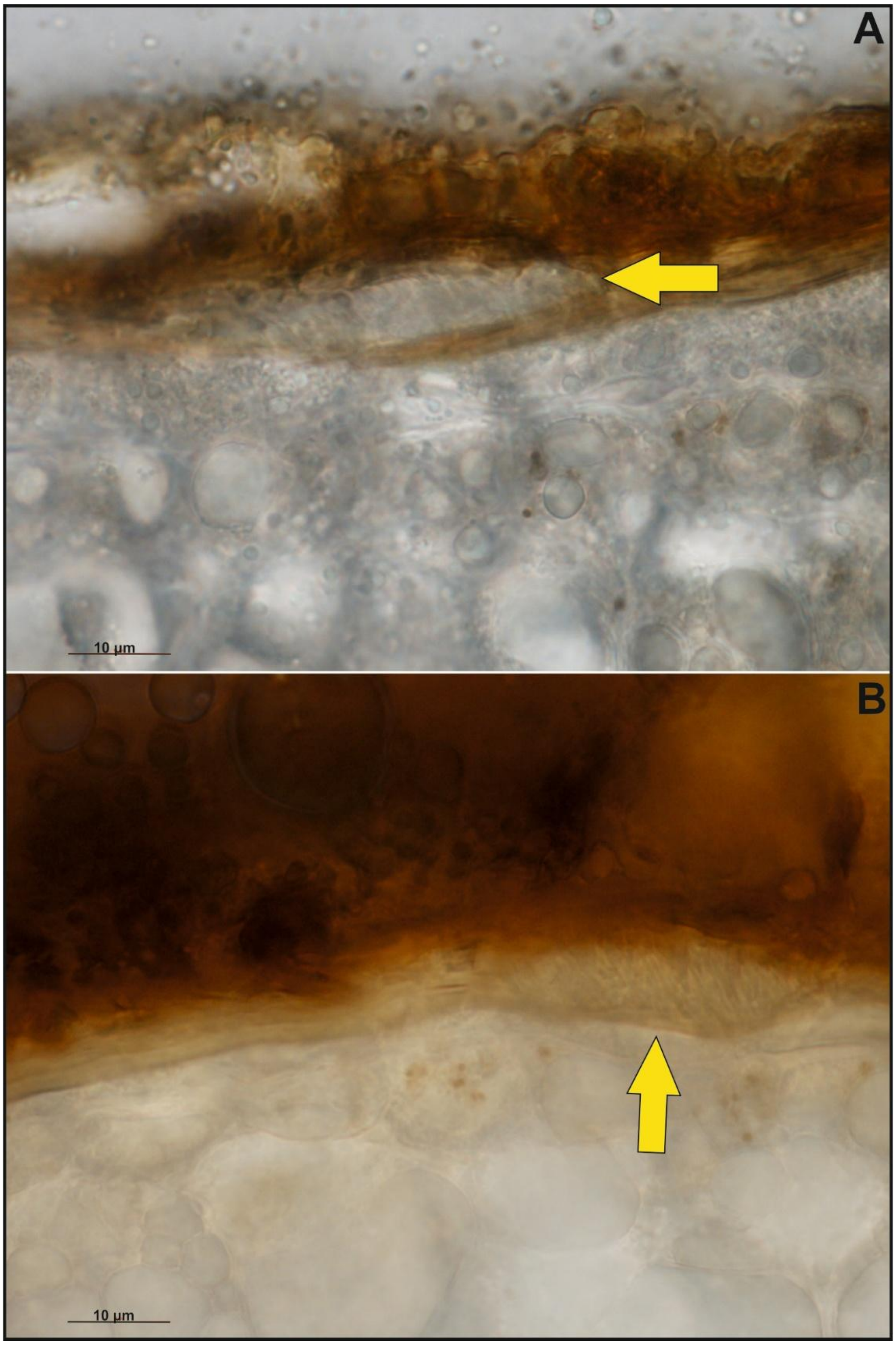

Figure S16. A-B) Crystal packets in cross-section within *Impatiens radiata* seeds, visualized under a light microscope at varying magnifications; indicated by yellow arrows.

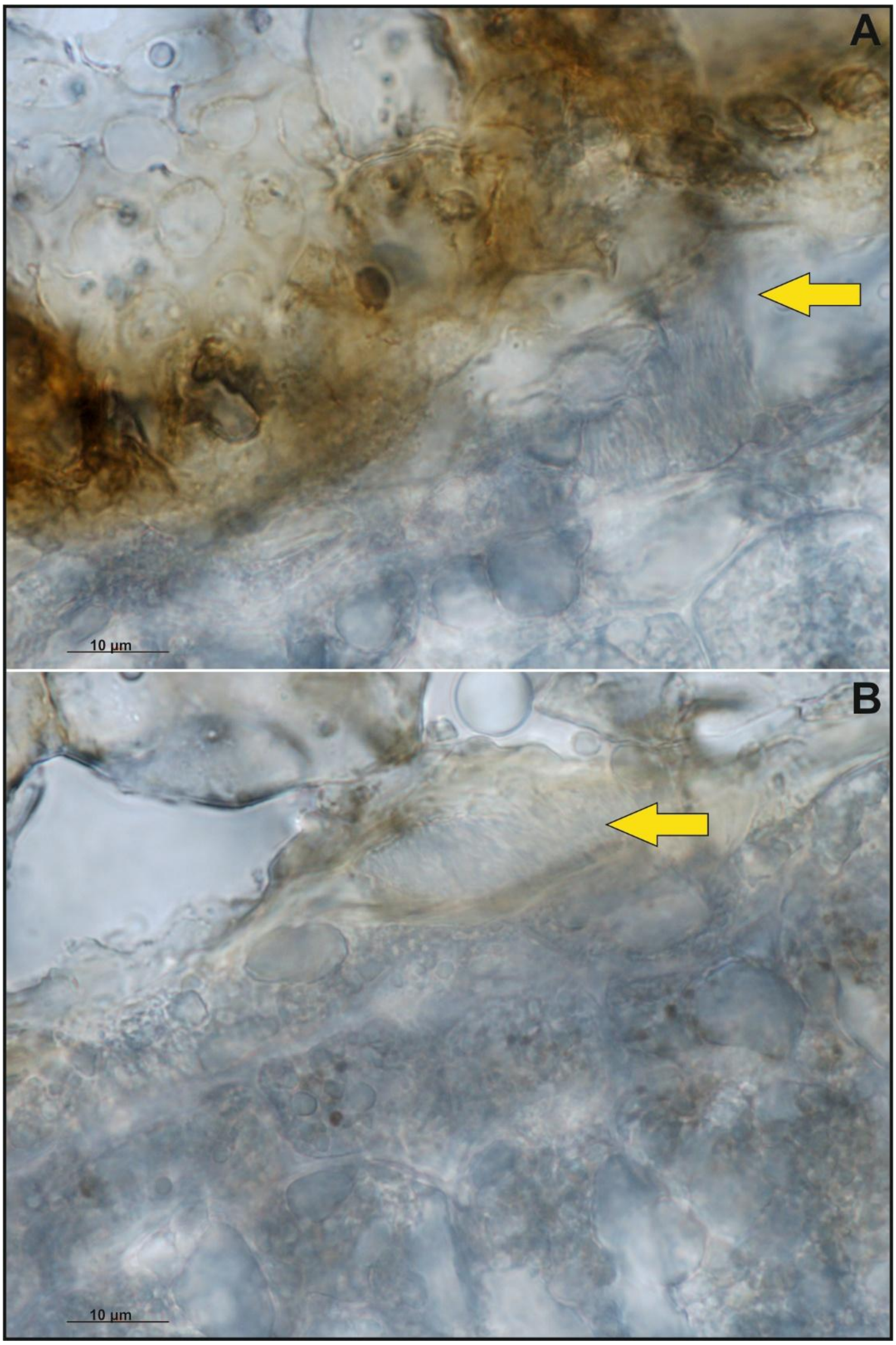

Figure S17. A-B) Crystal packets in cross-section within *Impatiens longiloba* seeds, visualized under a light microscope at varying magnifications; indicated by white arrows.

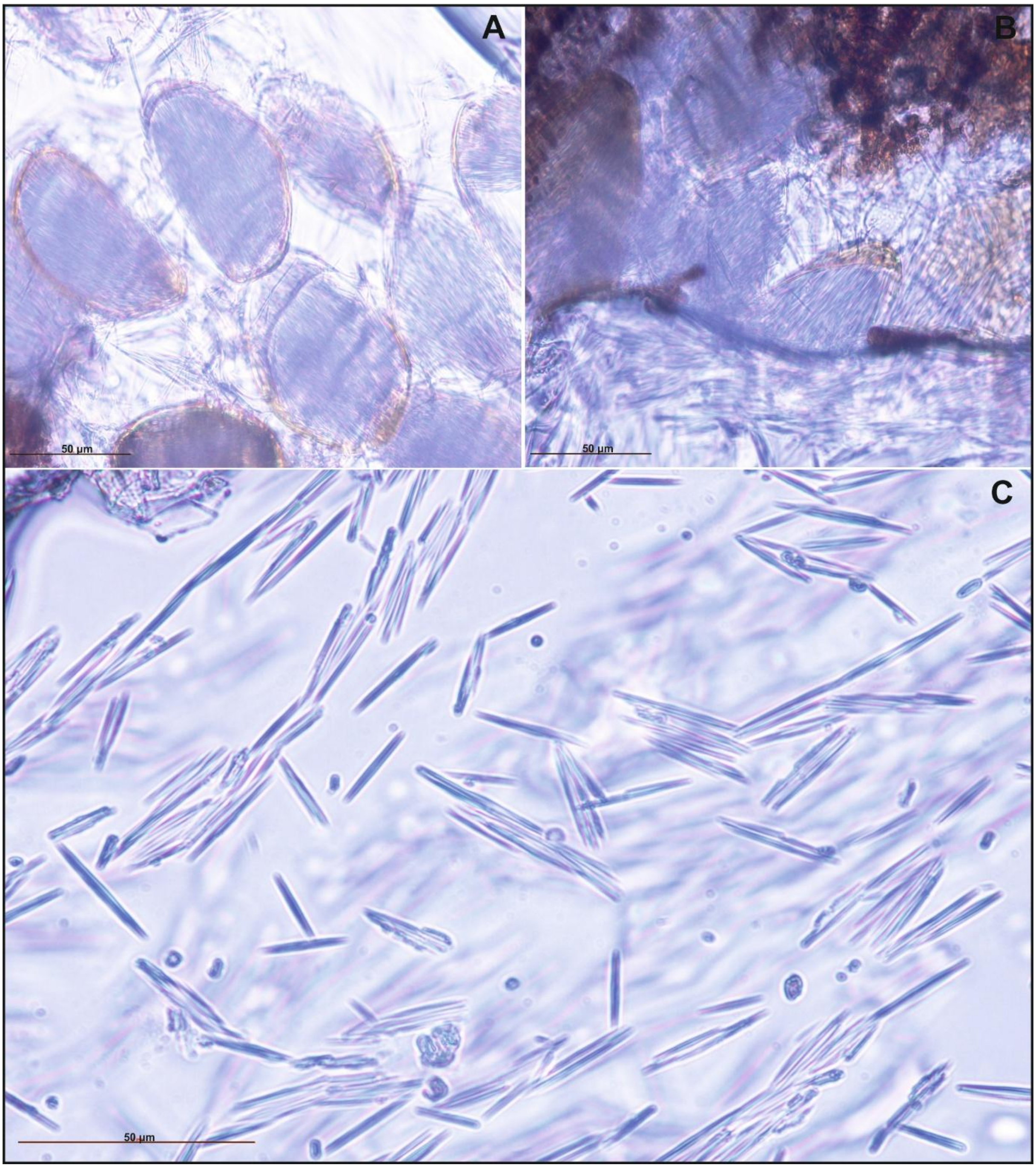

Figure S18. A-C) A packet of crystals observed under a light microscope in *Impatiens kanburiensis* seeds observed at different magnifications.

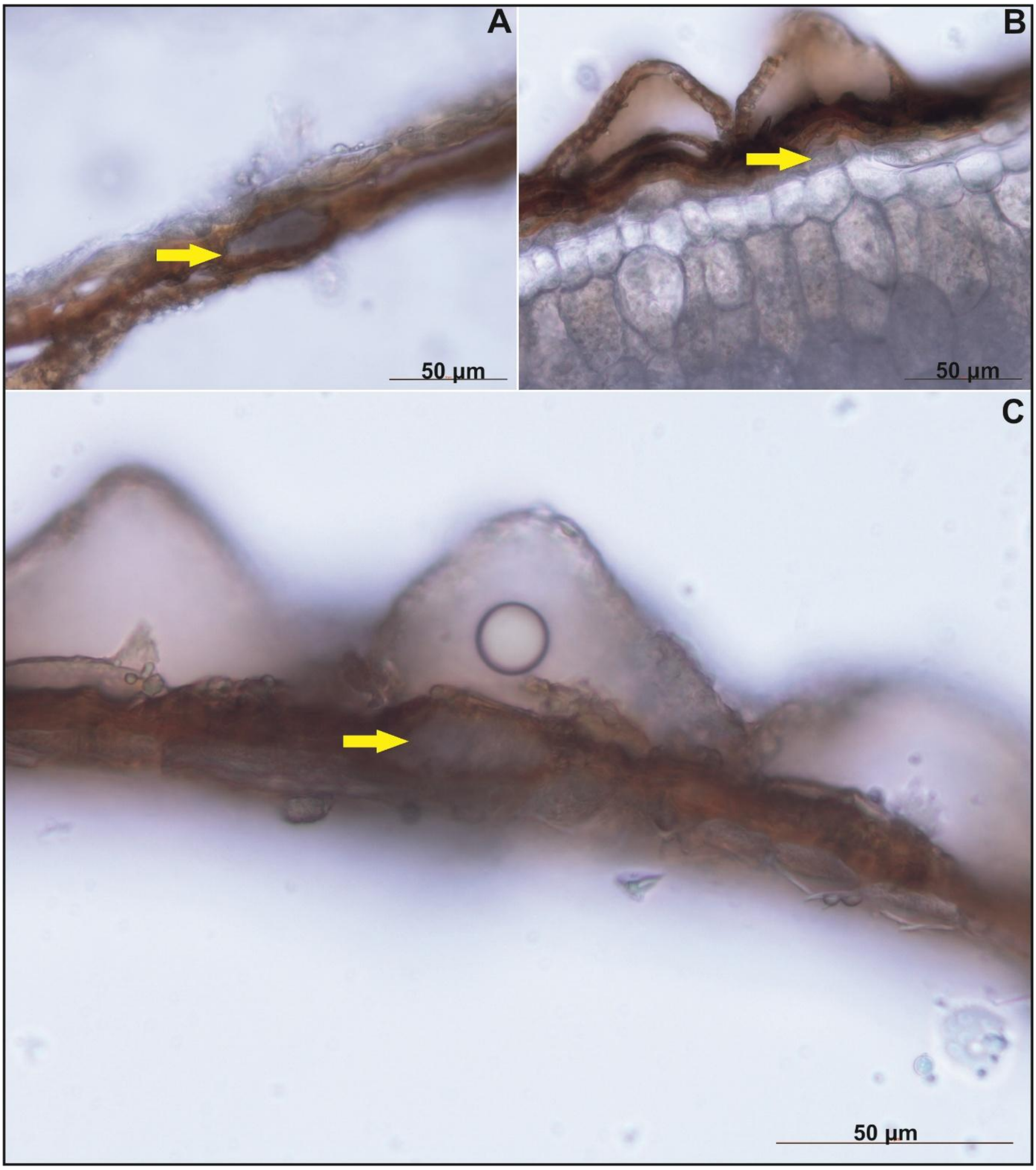

Figure S19. A-C) A packet of crystals observed under a light microscope in *Impatiens parviflora* seeds observed at different magnifications.

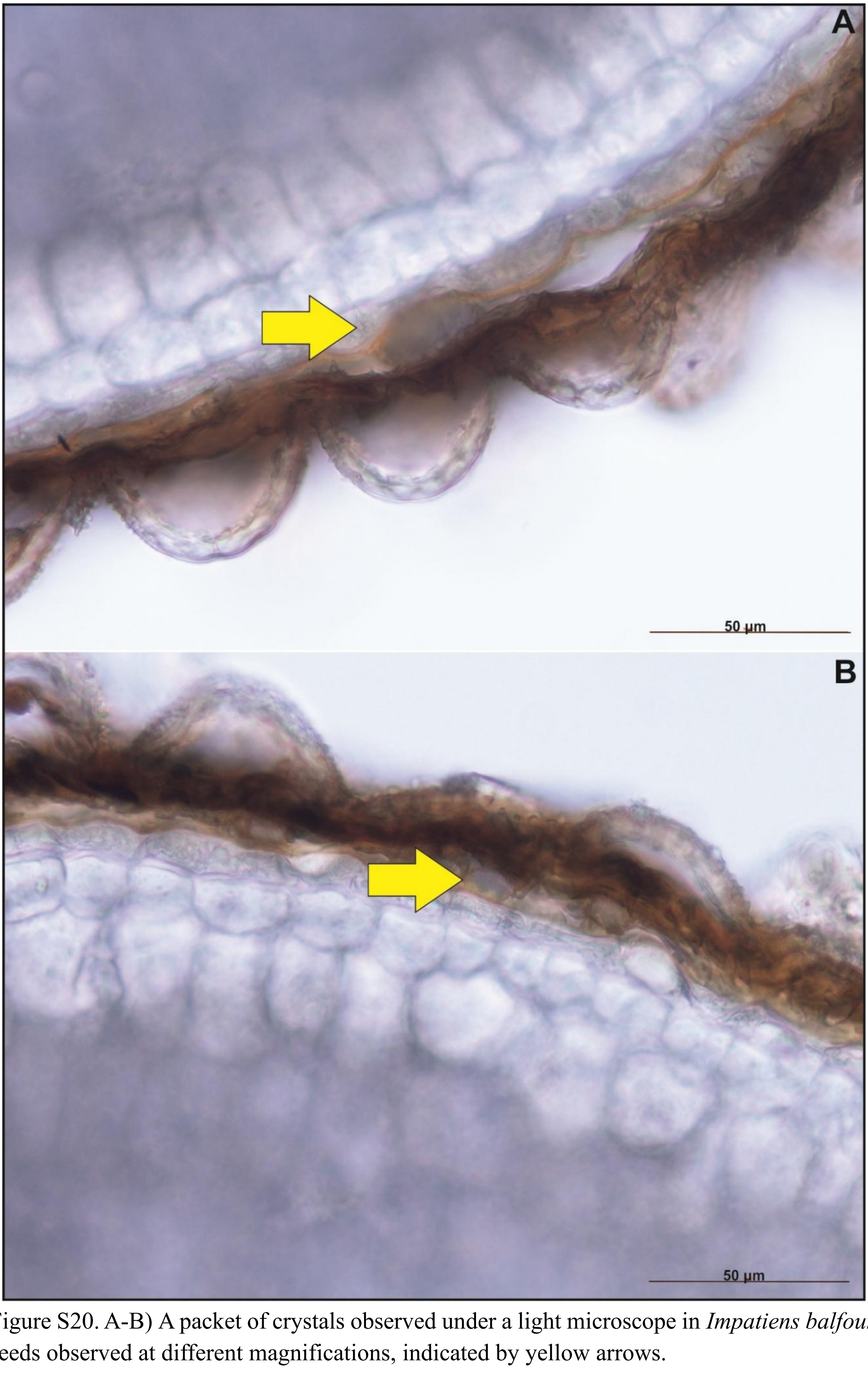

Figure S20. A-B) A packet of crystals observed under a light microscope in *Impatiens balfourii* seeds observed at different magnifications, indicated by yellow arrows.

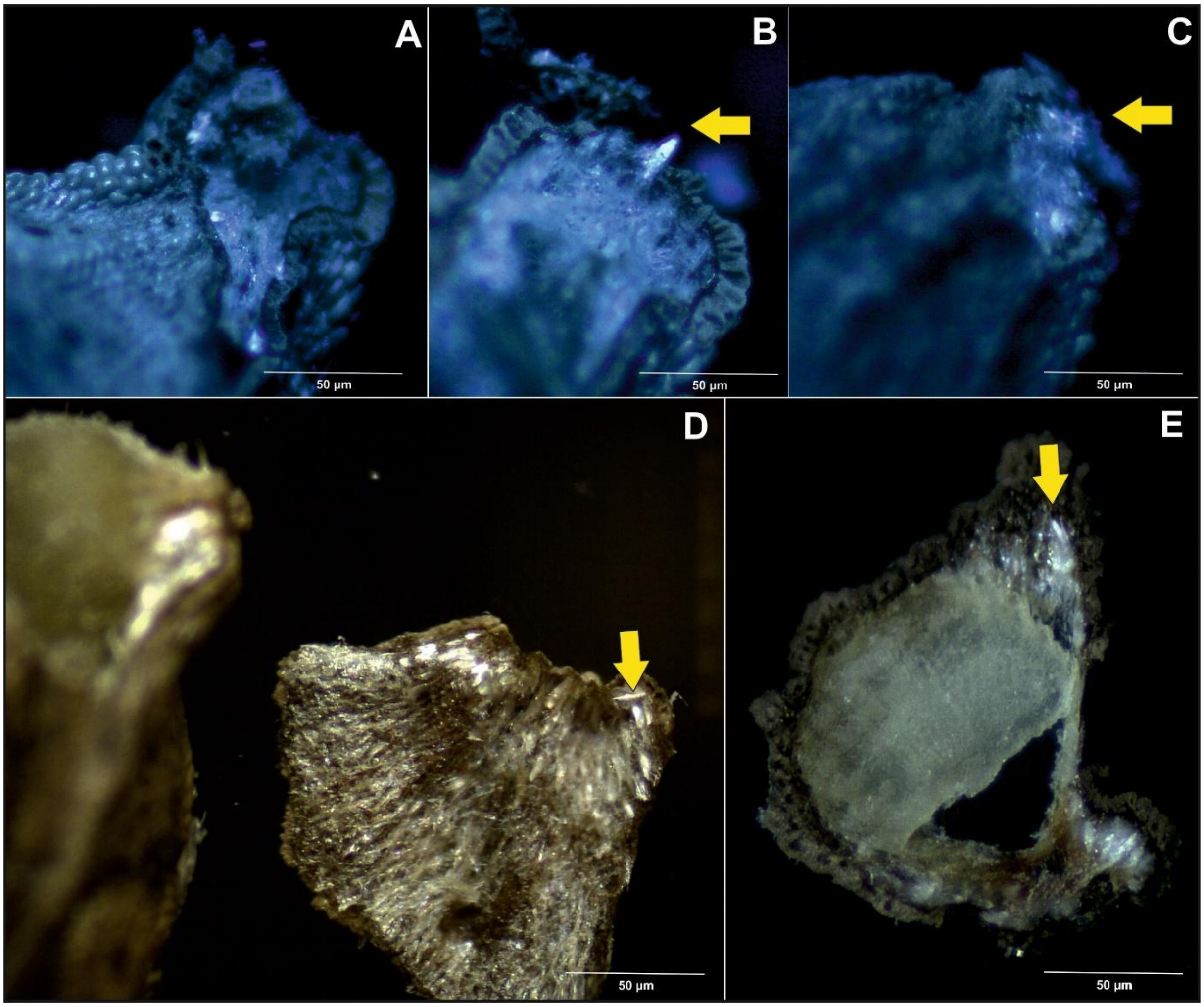

Figure S21. A–E) A packet of crystals in *Impatiens glandulifera* seed observed under a light microscope; marked with a yellow arrows.

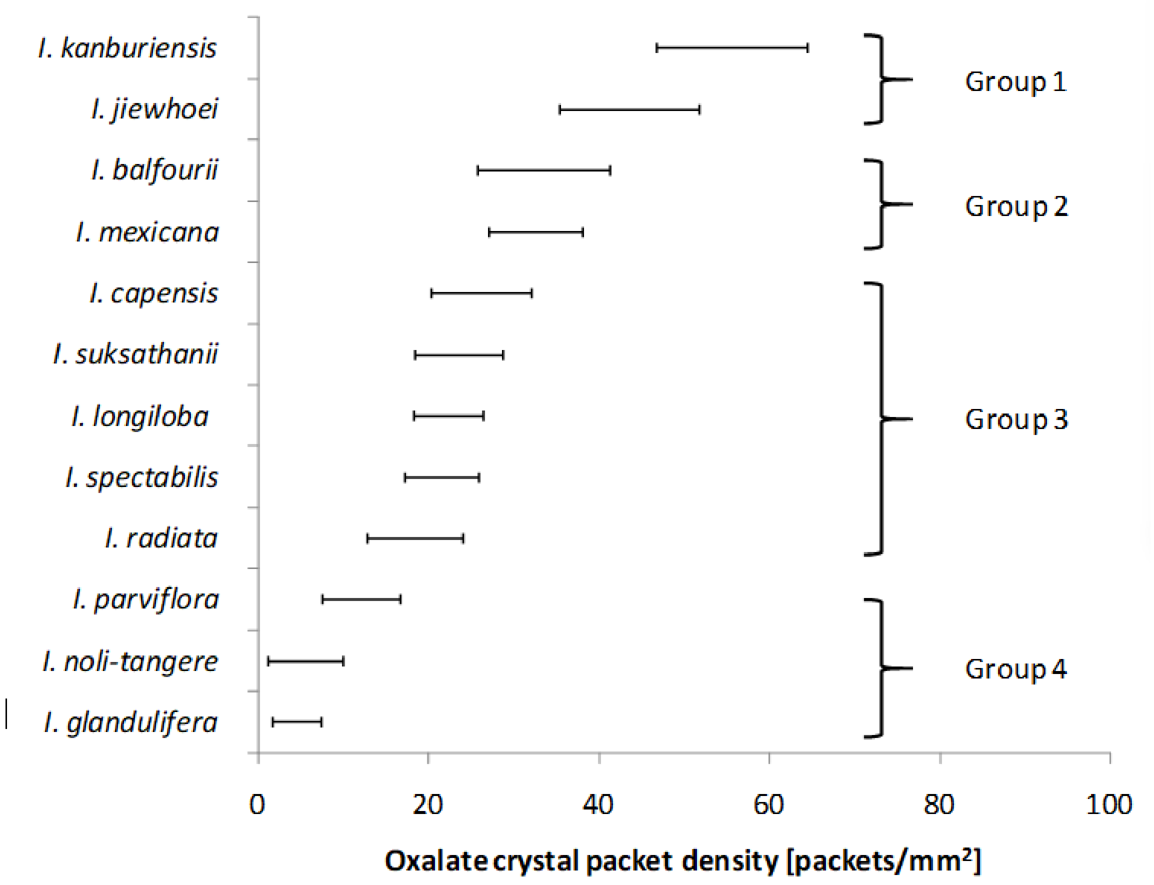

Figure S22. Tukey's confidence intervals ($p<0.05$) for oxalate crystal packet density in *Impatiens* species. Groups 1, 2, 3 and 4 include species with non-significant differences in density within groups and significant (at least $p<0.05$) differences between groups.
